# Brain-Inspired Hardware Solutions for Inference in Bayesian Networks

**DOI:** 10.3389/fnins.2021.728086

**Published:** 2021-12-02

**Authors:** Leila Bagheriye, Johan Kwisthout

**Affiliations:** Foundations of Natural and Stochastic Computing, Donders Institute for Brain, Cognition and Behaviour, Radboud University, Nijmegen, Netherlands

**Keywords:** brain inspired computing, Bayesian inference, spiking neural networks (SNN), nonvolatile, stochastic computing

## Abstract

The implementation of inference (i.e., computing posterior probabilities) in Bayesian networks using a conventional computing paradigm turns out to be inefficient in terms of energy, time, and space, due to the substantial resources required by floating-point operations. A departure from conventional computing systems to make use of the high parallelism of Bayesian inference has attracted recent attention, particularly in the hardware implementation of Bayesian networks. These efforts lead to several implementations ranging from digital circuits, mixed-signal circuits, to analog circuits by leveraging new emerging nonvolatile devices. Several stochastic computing architectures using Bayesian stochastic variables have been proposed, from FPGA-like architectures to brain-inspired architectures such as crossbar arrays. This comprehensive review paper discusses different hardware implementations of Bayesian networks considering different devices, circuits, and architectures, as well as a more futuristic overview to solve existing hardware implementation problems.

## Introduction

Bayesian inference (i.e., the computation of a posterior probability given a prior probability and new evidence; Jaynes, 2003) is one of the most crucial problems in artificial intelligence (AI), in areas as varied as statistical machine learning (Tipping, 2003; Theodoridis, 2015), causal discovery (Heckerman et al., 1999), automatic speech recognition (Zweig and Russell, 1998), spam filtering (Gómez Hidalgo et al., 2006), and clinical decision support systems (Sesen et al., 2013). It is a powerful method for fusing independent (possibly conflicting) data for decision-making in robotic, biological, and multi-sensorimotor systems (Bessière et al., 2008). Bayesian networks (Pearl, 1988) allow for a concise representation of stochastic variables and their independence and the computation of any posterior probability of interest in the domain spanned by the variables. The structure and strength of the relationships can be elicited from domain experts (Druzdzel and van der Gaag, 1995) or, more commonly, learned from data using algorithms such as expectation-maximization or maximum likelihood estimation (Heckerman et al., 1995; Ji et al., 2015). However, both the inference problem (Cooper, 1990) and the learning problem (Chickering, 1996) are NP-hard problems in general.

As a result, an efficient implementation of Bayesian networks is highly desirable. Although the implementation of inference on a large Bayesian network on conventional general-purpose computers provides high precision, it is inefficient in terms of time and energy consumption. Several complex floating-point calculations are required to estimate the probability of occurrence of a variable since the network is composed of various interacting causal variables (Shim et al., 2017). Moreover, the high parallelism feature of Bayesian inference is not used efficiently in conventional computing systems (F. Kungl et al., 2019). Conventional systems need exact values throughout the computation, preventing the use of the stochastic computing paradigm that consumes less power (Khasanvis et al., 2015a). To realize stochastic computing-based Bayesian inference especially using emerging nanodevices, it is highly needed to develop a robust hardware structure to overcome the characteristic imperfection of these new technologies. On the other hand, the practical realization and usage of large Bayesian networks has been problematic due to the abovementioned intractability of inference (Faria et al., 2021). Therefore, any hardware implementation of Bayesian inference needs to pay attention to a hierarchy of device, circuit, architecture, and algorithmic improvements.

Various approaches and architectures for Bayesian network hardware implementations have been developed; in the literature, approaches such as probabilistic computing platforms based on Field Programmable Gate Arrays (FPGAs), fully digital systems with stochastic digital circuits, analog-based probabilistic circuits, mixed-signal approaches, stochastic computing platforms with scaled nanomagnets, and Intel’s Loihi chip have been proposed.

In this overview paper, we describe these different approaches as well as the pros and cons of each of them. To this end, in *Section* “*Bayesian Networks and the Inference Problem*,” some basic preliminaries on Bayesian networks will be explained. *Section “Probabilistic Hardware-Based Implementation of Bayesian Networks”* describes several probabilistic neuronal circuits for Bayesian network variables using different nonvolatile devices. We explain neural sampling machines (NSMs) for approximate Bayesian inference. In *Section “New Computing Architecture With Nonvolatile Memory Elements for Bayesian Network Implementation*,” different systems for the implementation of Bayesian networks will be discussed that make use of new nonvolatile magnets and CMOS circuit elements. *Section “Bayesian Inference Hardware Implementation With Digital Logic Gates”* explains digital implementations of Bayesian inference algorithms as well as the definition of a standard cell-based implementation. At the end of this section, probabilistic nodes based on CMOS technology will be discussed. *Section “Crossbar Arrays for Bayesian Networks Implementation”* represents two brain-inspired hardware implementations of naïve Bayesian classifiers in the crossbar array architecture, in which memristors are employed as nonvolatile elements for algorithm implementation. Also, Bayesian reasoning machines with magneto-tunneling junction-based Bayesian networks are described. In *Section “Bayesian Neural Networks*,” employing Bayesian features in neural networks is represented. First Bayesian neural networks are explained. Then, Gaussian synapses for probabilistic neural networks (PNNs) will be introduced. Afterward, PNN with memristive crossbar circuits is described. Approximate computing to provide hardware-friendly PNNs and an application of probabilistic artificial neural networks (ANNs) for analyzing transistor process variation are explained. In *Section “Hardware Implementation of Probabilistic Spiking Neural Networks*,” employing Bayesian features in Spiking Neural Network (SNN) is represented. The feasibility of nonvolatile devices as synapses in SNNs architectures will be discussed for Bayesian-based inference algorithms. A scalable sampling-based probabilistic inference platform with spiking networks is explained. Then, a probabilistic spiking neural computing platform with MTJs is explained. Afterward, high learning capability of a probabilistic spiking neural network implementation and hardware implementation of SNNs utilizing probabilistic spike propagation mechanism are described. At the end of this section, memristor-based stochastic neurons for probabilistic SNN computing and Loihi based Bayesian inference implementation are represented. In *Section “Discussion*,” we provide an overall discussion of the different approaches. Finally, *Section “Conclusion”* concludes the paper.

## Bayesian Networks and the Inference Problem

A discrete joint probability distribution defined over a set of random (or stochastic) variables assigns a probability to each joint value assignment to the set of variables; this representation, as well as any inference over it, is exponential in the number of variables. For most practical applications, however, there are many independences in the joint probability distribution that allows for a more concise representation. There are several possible ways to represent such independences in probabilistic graphical models, representing a probabilistic model with a graph structure (Korb and Nicholson, 2010). The commonly described graphical models are Hidden Markov Models (HMMs), Markov Random Fields (MRFs), and Bayesian networks. MRFs use undirected graphs to represent conditional independences and capture stochastic relations in potentials. Bayesian networks use directed a-cyclic graphs, capturing stochastic relations in conditional probability tables (CPTs). Both structures can represent different subsets of conditional independence relations. HMMs are dynamic Bayesian networks that efficiently model endogenous changes over time, under the assumption of the Markov property.

**FIGURE 1:**
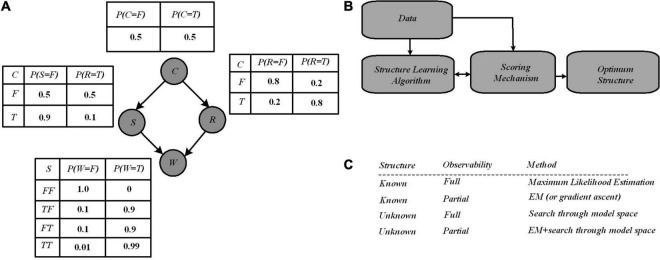
**(A)** A Bayesian network with four variables where independence between variables has been reported *via* conditional probability tables (CPTs). All posterior probabilities of interest in this network can be computed using the laws of probability theory, notably Bayes’ rule (1) that allows inferring the causes of effects observed in the network. **(B)** Structure learning flow in Bayesian networks through which Learning algorithm provides graphs from data, and are scored by the scoring mechanism. Finally, an optimum structure is selected after iteratively improvements of the score over the graph structure (Kulkarni et al., 2017b). **(C)** Several learning case in Bayesian networks (Murphy, 1998).

A simple Bayesian network with four variables (Pearl, 1988) has been shown as a running example in Figure 1 in which Bayesian networks are represented by a directed acyclic graph composed of nodes and edges and a set of CPTs. The nodes in the graph model random variables, whereas the edges model direct dependencies among the variables.

The four binary variables (denoted True or False, or equally “1” or “0”) “C,” “R,” “S,” and “W” represent whether it is cloudy, it is rainy, the sprinkler is on, and the grass is wet, respectively. The conditional probabilities (given in the CPTs) describe the conditional dependencies between parent and child nodes. Based on the network structure, the inference operation estimates the probability of the hidden variables, based on the observed variables (Pearl, 1988). For example, suppose one observes that the grass is wet, then the inference operation seeks to compute the probability distribution over the possible causes. There are possibly two hidden causes for the grass being wet: if it is raining or the sprinkler is on. Bayes’ rule defined in Equation (1), is used to calculate the posterior probability of each cause when wet grass has been observed; it allows us to compute this distribution from the parameters available in the CPTs:


(1) $$\text{P}\left(\text{S}|\text{W}\right)=\frac{\text{P}\left(\text{W}|\text{S}\right)\text{P}\left(S\right)}{\text{P}\left(W\right)}$$

For data analysis, the graphical model provides several benefits. Different methods are utilized for data analysis, which are rule bases, decision trees, and ANNs. Different techniques for data analysis are density estimation, classification, regression, and clustering. Then, what do Bayesian methods provide? One, it readily handles the missing of some data entries since the model encodes dependencies among all variables. Two, a Bayesian network paves the way to understanding about a problem domain and predicting the consequences of intervention *via* learning the causal relationships. Three, the model provides a causal and probabilistic semantics, though which an ideal representation for combining prior knowledge and data is possible. Four, with Bayesian statistics as well as Bayesian networks, the overfitting of data can be solved (Heckerman, 2020).

Learning a Bayesian network has two major aspects, i.e., discovering the optimal structure of the graph and learning the parameters in the CPTs. Learning a Bayesian network from data requires two steps of structure learning and parameter learning. There are a few works focusing on hardware implementation for structure learning. In order to find an optimal structure, exploring all possible graph structures for a given dataset is necessary. As shown in Figure 1B, for structure learning, based on the data, an algorithm starts with a random graph, then a scoring mechanism determines how well the structure can explain the data, where this quality is typically a mix of simplicity and likelihood. The graph structure is updated based on the score, and as a graph provides a better score, it is accepted. Several algorithms have been proposed in the literature for structure learning, with the two major scoring mechanisms being Bayesian scoring and information-theoretic scoring (Kulkarni et al., 2017b). Most of the information-theoretic scoring methods are analytical, and then complex mathematical computations are required. These methods are currently performed by software and the required time for structure learning is impacted significantly. Equation (2) represents the Bayesian scoring that uses the Bayes’ rule to compute the quality of a given Bayesian network structure. Using the Bayes rule, for a given data, and for a structure, the Bayes score is defined by:


(2) P(structure|Data)∝P(Data|structure)×P(Data)

The score of a structure as shown by Equation (2) is proportional to how closely it can describe observed data and on the prior probability of the structure (which could be uniform or provided by a domain expert). The Bayesian score is calculated *via* stochastic sampling through which a model of the graph is generated with the CPT values set, and sampling over each node for several iterations is performed. For example, for a probability value of 0.5 for a node, with 10 sampling iterations, it is expected to show “True” in 5 iterations. To calculate the Bayesian score of the graph (i.e., defining the correlation degree between the sampled data and learning data), the inference data taken from the stochastic sampling process are utilized. Once the structure of the network has been learned from the data, parameter learning (i.e., using data to learn the distributions of a Bayesian network) can be performed efficiently by estimating the parameters of the local distributions implied by the structure obtained in the previous step. There are two main approaches to the estimation of those parameters in literature: one based on maximum likelihood estimation and the other based on Bayesian estimation (Heckerman, 2020). Parameter estimation still could be challenging when the sample sizes are much smaller than the number of variables in the model. This situation is called “small n, large p,” which brings a high variability unless particular care is taken in both structure and parameter learning. As mentioned above, the graph topology (structure) and the parameters of each CPT can be inferred from data. However, learning structure is in general much harder than learning parameters. Also, learning when some of the nodes are hidden, or in case data are missing, is harder than when everything is observed. This gives rise to four distinct cases with increasing difficulty shown in Figure 1C.

Bayesian network learning involves the development of both the structure and the parameters of Bayesian networks from observational and interventional datasets; Bayesian inference on the other hand is often a follow-up to Bayesian network learning and deals with inferring the state of a set of variables given the state of others as evidence. The computation of the posterior probabilities shown above (Figure 1A) is a fundamental problem in the evaluation of queries. This allows for diagnosis (computing P(cause| symptoms)), prediction (computing P(symptoms| cause)), classification (computing P(class| data)), and decision-making when a cost function is involved. In summary, Bayesian networks allow for a very rich and structured representation of dependencies and independencies within a joint probability distribution. This comes at the price of the intractability of both inference (i.e., the computation of posterior probabilities conditioned on some observations in the network) and learning (i.e., the establishment of the structure of the model and/or the conditional probabilities based on data and a learning algorithm). One can deal with this intractability either by reducing the complexity of the model or by accepting approximate results. Examples of the former are reducing the tree width of the network model (Kwisthout et al., 2010), reducing the structure of the model to a polytree describing a hidden state model and observable sensors (Hidden Markov model) (Baum and Petrie, 1966), or assuming mutual independence between features (Naïve Bayesian classifiers) (Maron and Kuhns, 1960). Examples of the latter are approximation algorithms such as Metropolis-Hastings (Hastings, 1970) and Likelihood weighting (Shachter and Peot, 1990).

## Probabilistic Hardware-Based Implementation of Bayesian Networks

This section represents several probabilistic neuron circuits for Bayesian network variables by using different nonvolatile devices connected to CMOS circuit elements. To this end, the first two abstraction layer-based implementations and then a direct implementation of probabilistic circuits will be discussed. Then, a NSM for approximate Bayesian inference is explained.

### Probabilistic Spin Logic-Based Implementation of Bayesian Networks

**FIGURE 2:**
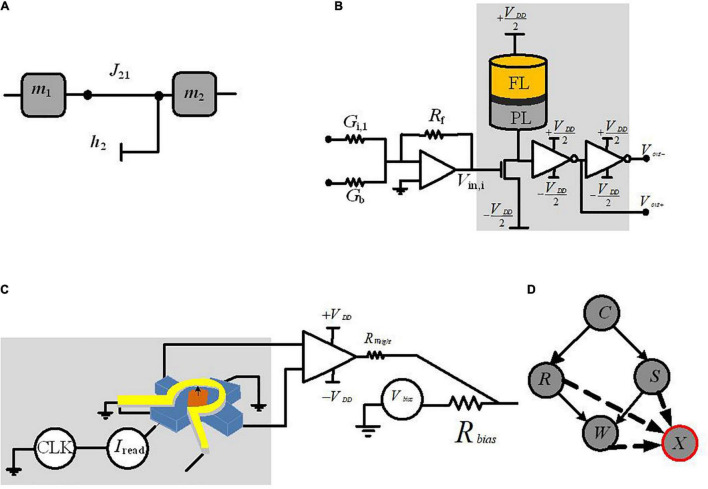
Circuit implementation of a p-bit block. **(A)** PSL-based representation of two-node Bayesian network. **(B)** The p-bit design based on MTJ p-circuit with connection weight and bias to be connected to another node (Faria et al., 2018). **(C)** The p-bit design, based on nanomagnet p-circuit with connection weight and bias to be connected to another node (Debashis et al., 2020). **(D)** The required auxiliary node, *X*, for representing the four-node Bayesian network.

The first approach we will discuss is the mapping of CPTs to probabilistic circuits constructed by probabilistic bits (p-bits) (Faria et al., 2018; Debashis et al., 2020). In this approach, each variable in a Bayesian network is modeled by a stochastic circuit, representing a specific conditional probability. This probability is represented by the input that comes from its parent nodes, *via* the weights of the links between nodes. For the p-bit implementation, the Bayesian network is translated into probabilistic spin logic (PSL). PSL is a behavioral model, represented by biasing (*h*) and interconnection (*J*) coefficients (shown in Figure 2A). Then, PSL is translated into electronic devices.

The reported p-bit implementation in Faria et al. (2018) as shown in Figure 2B uses a stochastic spintronic device, i.e., magnetic tunnel junction (MTJ), connected to the drain of a transistor.

Table 1 reports the required equations for the PSL translation into a circuit whose output *m*_1_ is related to its input *I*_2_ (the synapse generates the input *I*_2_ from a weighted state of *m*_2_, Figure 2A), Equation (3). Based on Equation (4A), a random number generator (RNG) (rand) and a tunable element (tanh) construct *m*_2_. The RNG is the MTJ and the tunable component is the NMOS transistor; *r*_MTJ_ is a correlated RNG and the NMOS transistor resistance *r*_T_ is approximated as a tanh function found by fitting based on *I*–*V* characteristics. The PSL model is then translated into electronic components (shown in Figure 2B) where each node (represented by *m*) is connected to other nodes and biased through voltages *V*_bias_ and conductances *G*; *V*_0_ is a fitting parameter. Biasing (*h*) and interconnection (*J*) coefficients of PSL model have been reported in Table 1 by Equations (5A)–(7A), due to its corresponding P-bit circuit in Figure 2B.

**TABLE 1:** PSL to circuit translation requirements.

*PSL elements*	*P-bit design-1* (Faria et al., 2018)		*P-bit design-2* (Debashis et al., 2020)

*PSL*		*I*_2_ = *J*_21_*m*_1_ + *h*_2_ (3)	
*Given CPT: m*_1_ = 0, *p*(*m*_2_ = 1) = *a m*_1_ = 1, *p*(*m*_2_ = 1) = *b*	*m*_2_(*t* + Δ*t*)=*sgn*(−*r*_MTJ_(*t* + Δ*t*) +*r*_T_(*t* + Δ*t*)) (4A)		*m*_2_ = σ(*I*_2_)=σ(*J*_21_*m*_1_ + *h*_2_) (4B)
*J*_21_	$J_{21}=\frac{G_{21}}{G_{b}}$ (5A)		$J_{21}=\pm \mu_{0}\frac{V_{\text{DD}}}{2}B_{0}R_{\text{weight}}$ (5B)
*h*_2_	$h_{2}=\frac{V_{\mathrm{bias},2}}{\frac{V_{\text{DD}}}{2}},$ (6A)		$h_{2}=\pm \mu_{0}\frac{V_{bias}}{2}B_{0}R_{bias}$ (6B)
*I*_2_	$I_{2}=\frac{V_{\mathrm{in},2}}{V_{0}}$ (7A)		$I_{2}=\pm \left(\mu_{0}\frac{V_{\text{DD}}}{2}B_{0}R_{\text{weight}}\right)m_{1}+\left(\mu_{0}\frac{V_{\text{bias}}}{2}B_{0}R_{\text{bias}}\right)$ (7B)

Note that individual p-bits require sequencers in software implementations to be programmed in a directed order. The p-bit in Faria et al. (2018) and Faria et al. (2021) is an autonomous, asynchronous circuit that can operate correctly without any clocks or sequencers, in which the individual p-bits need to be carefully designed and the interconnect delays, from one node to another node, must be much shorter than the nanomagnet fluctuations of the stochastic device. This condition is met as magnetic fluctuations occur at approximately the 1-ns time scale. However, in asynchronous operations, updating the network as well as dealing with variations in the thermal barriers or interconnect delays necessitates further study.

Debashis et al. (2020) present another alternate p-bit implemented with inherently stochastic spintronic devices based on a nanomagnet with perpendicular magnetic anisotropy. This device utilizes the spin orbit torque from a heavy metal (HM) under-layer to be initialized to its hard axes. Equations (4B)–(7B) in Table 1 show the relation between the stochastic variables *m*_1_ and *m*_2_ based on the corresponding p-bit circuit in Figure 2C. Here, σ defines the sigmoidal activation function for the device in *m*_2_. Equation (4B) explains the conditional dependencies. The probability of *m*_2_ being 1 given *m*_1_ being 1 is calculated through Equation (4B) while setting *m*_1_ = 1. The parameters *B*_0_ and *h*_2_ represented in Equations (5B)–(7B) can be tuned to change the shape and offset of the sigmoidal activation function (while presenting the CPTs *via* the connection weights).

To implement the four-node Bayesian network by p-bits, Figure 2D, using PSL behavioral models in Faria et al. (2018) and Debashis et al. (2020), requires an auxiliary p-bit defined by node “*X.*” The CPT of node “*W*” has four conditional probability distributions (based on nodes “*R*” and “*S*,” see Figure 1); based on the principles of linear algebra, this CPT needs four independent parameters. The interconnection weights *J*_WR_ and *J*_*WS*_ and the bias parameter to the node “*W*” (*h*_W_) are three parameters of four. The fourth parameter has been implemented with the interconnection to node “*X.*” Nodes with *N* parents need a total of (*N*+ 1) parameters and 2*N* equations to meet the PSL model requirement. Based on the number of linearly independent equations, it is needed to represent the appropriate number of auxiliary variables (Faria et al., 2018). Utilizing the auxiliary nodes in p-bit-based implementation of Bayesian networks adds extra area/energy overhead and requires further studies.

### Spintronic Devices for Direct Hardware Implementation of Bayesian Networks

**FIGURE 3:**
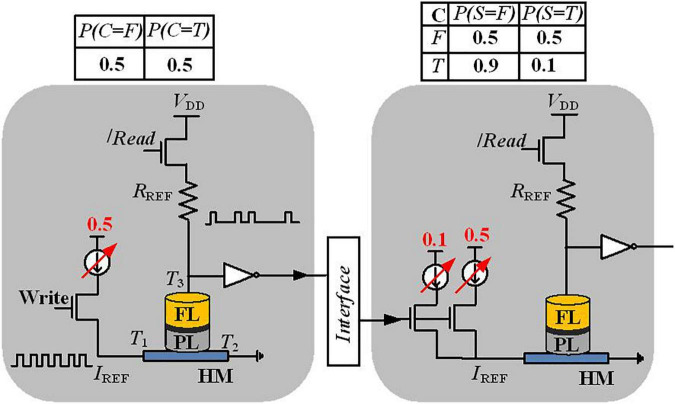
Detailed implementation view of a Bayesian network variable and the interconnection between the two nodes C and S in the four-node Bayesian network of Figure 1.

In Shim et al. (2017), a direct implementation of Bayesian networks has been proposed with a stochastic device that is based on a three-terminal device structure, shown in Figure 3. The proposed stochastic device can be developed by fabricating an MTJ stacked on top of the ferromagnet-HM layers. The stochastic switching of the device in the presence of thermal noise has been employed to implement a Bayesian network. This MTJ with two permanent states (represent two different resistance levels) models stored values by the resistance levels (Shim et al., 2017). The MTJ is composed of a tunneling barrier (TB) sandwiched between two ferromagnetic layers, namely, the free layer (FL) and the pinned layer (PL). The relative magnetization direction of two ferromagnetic layers defines the MTJ state; MTJ shows low (or high) resistance when the relative magnetization direction is parallel (or anti-parallel) (Bagheriye et al., 2018). Based on the write current through terminals *T*_1_ and *T*_2_, which probabilistically switches the magnet (with a probability controlled by the current magnitude), the read path through the terminals *T*_3_ and *T*_2_ controls the final state of the magnet.

In order to represent a variable of the Bayesian network, a Poisson pulse train generator translates the probability data into the frequency of the output pulses. Thanks to the controllable stochastic switching of the nanomagnet, along with current sources and some circuit elements [reference resistor (*R*_ref_) and separate write and read paths], the Poisson spikes can be generated as shown in Figure 3. A reference resistor is used to generate a Poisson spike train, where the number of spikes encodes information about the probability. For instance, if 30 spikes are observed at the output of the “S” node in 100 write cycles, this determines that the probability of “S is True” is 30%. Moreover, for more complex inference, extra arithmetic building blocks using CMOS circuits between two Poisson pulses are needed.

### Neural Sampling Machine for Approximate Bayesian Inference

**FIGURE 4:**
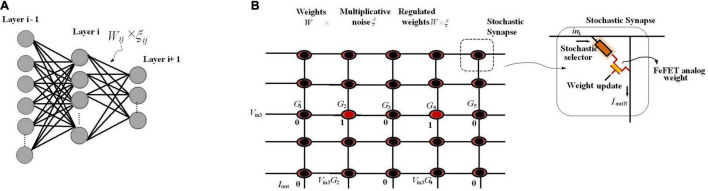
An NSM implemented in hardware using crossbar array architecture. **(A)** The utilized NSM with an input layer, three hidden layers, and an output layer. **(B)** The stochastic selector device used for injecting Bernoulli multiplicative noise is placed at each cross-point connected to an analog weight cell implemented using eNVMs. The stochastic selector element provides the selectively sampling or reading the synaptic weights *G*_ij_ with some degree of uncertainty controlled by random binary variables ξ_ij_.

In biological neural networks, synaptic stochasticity occurs at the molecular level, and due to the presynaptic neuronal spike, the neurotransmitters at the synaptic release site release with a probability of approximately 0.1. Dutta et al. (2021) presented a neuromorphic hardware framework to support a recently proposed class of stochastic neural networks called the neural sampling machine (NSM), which mimics the dynamics of noisy biological synapses. NSM incorporates a Bernoulli or “blank-out” noise in the synapse to being multiplicative, which has an important role in learning and probabilistic inference dynamics. This performs as a continuous DropConnect mask on the synaptic weights, where a subset of the weights is continuously forced to be zero. Stochasticity is switched off during inferencing in DropConnect, whereas it is always on in an NSM providing probabilistic inference capabilities to the network. Figure 4 shows the hardware implementation of NSM using hybrid stochastic synapses. These synapses consist of an embedded non-volatile memory, eNVM [a doped HfO2 ferroelectric field-effect transistor (FeFET)-based analog weight cell] in series with a two-terminal Ag/HfO2 stochastic selector element. By changing the inherent stochastic switching of the selector element between the insulator and the metallic state, the Bernoulli sampling of the conductance states of the FeFET can be performed. Moreover, the multiplicative noise dynamics has a key side effect of self-normalizing, which performs automatic weight normalization and prevention of internal covariate shift in an online fashion. The conductance states of the eNVM in the crossbar array (which performs row-wise weight update and column-wise summation operations in a parallel fashion) are adapted by weights in the Deep Neural Network (DNN). In order to implement an NSM with the same existing hardware architecture, selectively sampling or reading the synaptic weights Gij with some degree of uncertainty is required. A selector device as a switch has been employed, stochastically switching between an ON state (representing ξij = 1, ξij generated for each of the synapse and is a random binary variable) and an OFF state (ξij = 0). Figure 4B depicts an input voltage *V*_in3_ applied to a row of the synaptic array with conductance states G = {G_1_, G_2_, G_3_, G_4_,…, G_N_}, and based on the state of the selectors in the cross-points, an output weighted sum current *I*_out_ = {0, G_2_.*V*_in3_, 0, G_4_.*V*_in3_, 0} is obtained, which is exactly the same as multiplying the weight sum of *W*_ij_*Z*_j_ (*Z*_j_, is the activation function of the neuron j) with a multiplicative noise ξ_ij_.

For Bayesian inference, the hardware NSM captures uncertainty in data and produces classification confidence. To this end, in Dutta et al. (2021), the hardware NSM has been trained on the full MNIST dataset. During the inference mode, the performance of the trained NSM on continuously rotated images has been evaluated where, for each of the rotated images, 100 stochastic forward passes are performed and the softmax input (output of the last fully connected hidden layer in Figure 4A) as well the softmax output were recorded. The NSM will correctly predict the class corresponding to an input neuron if the softmax input of a particular neuron is larger than all the other neurons. However, as the images are rotated more, even though the softmax output can be arbitrarily high for, e.g., neuron 2 or 4 predicting that the image are 2 or 4, respectively, the uncertainty in the softmax output is high, showing that the NSM can account for the uncertainty in the prediction. The uncertainty of the NSM has been quantified by looking at the entropy of the prediction, defined as *H* = −∑_P_*log_(*P*)_, where *p* is the probability distribution of the prediction. When the NSM makes a correct prediction, the uncertainty measured in terms of the entropy remains 0. However, in the case of wrong predictions, the uncertainty associated with the prediction becomes large. This is in contrast to the results obtained from a conventional MLP network (deterministic neural network) where the network cannot account for any uncertainty in the data.

## New Computing Architecture With Nonvolatile Memory Elements for Bayesian Network Implementation

In this section, several Bayesian network implementation systems will be discussed that make use of new nonvolatile magnets and CMOS circuit elements. We will first explain FPGA-like architectures and then discuss developed spintronic-based inference systems.

### Direct Physical Equivalence Implementation of Bayesian Networks

**FIGURE 5:**
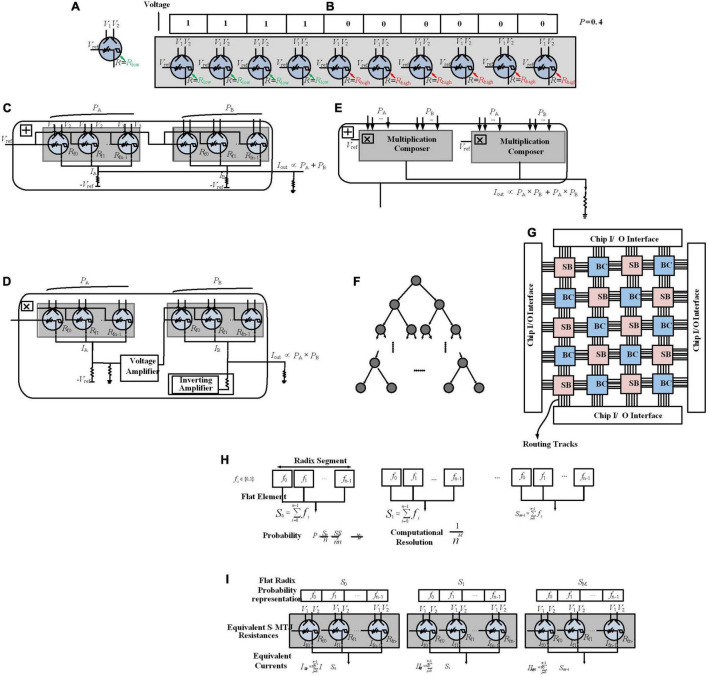
Probability encoding *via* Strain-switched Magneto-Tunneling Junction (S-MTJ) device. **(A)** A S-MTJ Device. **(B)** Probability data encoding by spatially distributed digits, and two-state S-MTJs for physically equivalent representations. Probability value *P* = 0.4 has been encoded with 10 digits (resolution of 0.1). Probability composer framework. **(C)** Addition composer. **(D)** Multiplication composer. **(E)** Add–Multiply composer. Physically Equivalent Architecture for Reasoning (PEAR). **(F)** Tree Bayesian Network. **(G)** Mapping every node in a Bayesian network graph to a Bayesian cell (BC) on PEAR. **(H)** Flat-Radix information representation pattern. Probability is encoded in segments where each segment has a radix arrangement and contains flat elements. **(I)** Probability data encoding by the proposed information representation scheme, and S-MTJs for physically equivalent representations.

In Khasanvis et al. (2015a), in addition to transistors, strain-switched magneto tunneling junctions (S-MTJs) are used for a Bayesian hardware implementation. S-MTJs as nonvolatile devices provide low switching energy (Atulasimha and Bandyopadhyay, 2013). As shown in Figure 5A, it has four terminals and the resistance between reference and output terminals can be changed by the two input digital voltage terminals change. It shows hysteresis in resistance vs. voltage characteristics that provides non-volatility. Khasanvis et al. (2015a) represents a mindset of physical equivalence, which means each digit in the probability representation is mapped directly (without any abstraction layer) to S-MTJ resistance with equivalent digital voltage representation (Figure 5B), while the proposed work in Faria et al. (2018) and Debashis et al. (2020) need the PSL abstraction level to map Bayesian networks in hardware.

For encoding, *n* spatially distributed digits *p*_1_, *p*_2_,…, *p*_*n*_ have been defined (Figure 5B), each digit *p*_i_ can be any one of *k* values, the number of states of the physical device (e.g., for devices with two states, *k* = 2 and *p*_i_ ∈ {0, 1}). The encoded probability *P* is defined by: *P* = $\frac{\sum_{i=1}^{n}p_{i}}{n\left(k-1\right)}$, which is called a flat linear representation (resolution is determined by the number of digits *n*). These digits have been physically represented in resistance domains using two-state S-MTJs, where *R*_low_ represents digit 1 and *R*_high_ represents digit 0. For Bayesian computations in hardware, it is necessary to have analog arithmetic functions such as probability addition and multiplication. Figures 5C–E depict arithmetic composers, which are operating intrinsically on probabilities as elementary building blocks. Figures 5C–E show the addition composer, multiplication composer, and add–multiply operation composer, respectively, as well as support circuits such as amplifiers, implemented with CMOS operational amplifiers.

In Khasanvis et al. (2015a), thanks to the analog arithmetic composers, a paradigm departure from the Von Neumann paradigm has been developed that uses a distributed Bayesian cell (BC) architecture. In this architecture, each BC maps a Bayesian variable in hardware as physical equivalence, shown in Figures 5F,G, named Physically Equivalent Architecture for Reasoning (PEAR). BCs are constructed from probability arithmetic composers and are used to include CPTs, likelihood vectors, belief vectors, and prior vectors; BCs locally store these values continuously and perform inference operation, removing the need for external memory (Khasanvis et al., 2015a). Metal routing layers are used for BC interconnection. This connectivity is programmable through reconfigurable switch boxes (SBs) (similar to FPGAs) to map arbitrary graph structures.

Bayesian networks using binary trees, as shown in Figures 5F,G, have been mapped directly in hardware on PEAR. This computing architecture scales the number of variables to a million. Although for a resolution of 0.1, it gains three orders of magnitude efficiency improvement in terms of runtime, power, and area over implementation on 100-core microprocessors, it does not support efficient scaling for higher resolutions. To increase the resolution, it is needed to change the abovementioned flat linear representation that increases area linearly (where a single probability value requires multiple physical signals). To this end, as shown in Figures 5H,I, another S-MTJ-based circuit paradigm leveraging physical equivalence with a new approximate circuit-style has been reported (Kulkarni et al., 2017a), where the computation resolution is 1/(*n*^*M*^) where *M* is the number of Radix segments where each segment is composed of flat elements. This is a new direction on scaling computational resolution, which is a hybrid method for representing probabilities, aiming to provide networks with millions of random variables. Here, precision scaling provides much lower power and performance cost than in Khasanvis et al. (2015b) for PEAR implementation *via* offering area overhead at a logarithmic vs. linear scale. Results show a 30× area reduction for a 0.001 precision vs. prior direction (Khasanvis et al., 2015a) while obtaining three orders of magnitude benefits over 100-core processor implementations.

### Stochastic Hardware Frameworks for Learning Bayesian Network Structure

**FIGURE 6:**
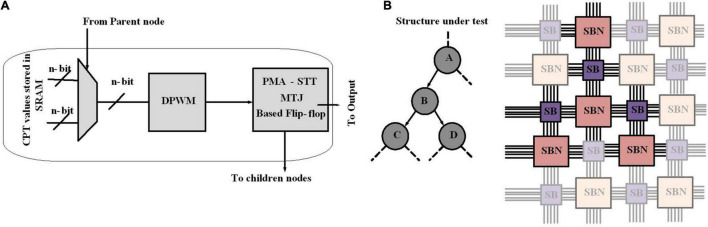
Design flow for structure learning in Bayesian networks. **(A)** Building blocks of Stochastic Bayesian Nodes (SBN). **(B)** Every node in a Bayesian network is mapped to an SBN reconfigurable framework. The links of the Bayesian network are implemented with metal routing layers and the connectivity programmed through switch boxes. Stochastic bitstream generator blocks for Bayesian network implementation.

A Bayesian network has two major aspects: the structure of the graph and the parameters in the CPT and determining the structure of a Bayesian network is known as structure learning. In Kulkarni et al. (2017b), the stochastic behavior of emerging magneto-electric devices is used to accelerate the structure learning process of Bayesian learning, which results in reducing the runtime by five orders of magnitude for Bayesian inference. For structure learning, based on the data, an algorithm starts with a random graph, then a scoring mechanism determines how well the structure can explain the data, where this quality is typically a mix of simplicity and likelihood. The graph structure is updated based on the score; as a graph provides a better score, it is accepted. To perform scoring, the framework should support mapping of arbitrary Bayesian networks; hence, configurability is necessary. The proposed design employs an FPGA-like reconfigurable architecture constructed from a set of programmable SBs and Stochastic Bayesian Nodes (SBNs). For scoring a Bayesian structure, nodes are mapped into SBN and the connectivity between nodes is implemented by SBs (Figures 6A,B).

Stochastic Bayesian Nodes represents a node in a Bayesian network. The node consists of multiplexers, a digital pulse width modulator (DPWM), and perpendicular magnetic anisotropy spin transfer torque magnetic tunnel junctions (PMA-STT MTJs). The switching operation of PMA-STT MTJ is probabilistic and directly controlled *via* modulating the duration of the applied current; this unique property has been employed to design circuits to perform probabilistic operations. As shown in Figure 6A, the CPT values are preconfigured in the SRAM cell. An appropriate CPT value to be sent to the DPWM is selected by multiplexers based on the output of the parent SBN. A DPWM generates voltage pulses with precise duration. Once the pulse corresponding to the CPT value is fed to the MTJ, the output is stored in a flip-flop. The output of the flip-flop is available for read-out and is also sent to the next node. The configured Bayesian structure is stochastically sampled to reach sufficient statistics. The sampled data are employed to calculate the Bayesian score of that structure, through Equation (2).

Through this hardware acceleration of the structure discovery (*via* scoring mechanism) process of Bayesian learning, the runtime for Bayesian network inference has been highly reduced (Kulkarni et al., 2017b). This property attracts more attention to structure learning acceleration and turns out to be a promising field to be studied.

### Stochastic Bitstream Generator Blocks for Bayesian Network Implementation

In Jia et al. (2018), the inherent stochastic behavior of spintronic device, MTJs, has been used to build a stochastic bitstream generator (SBG), which is critical for the Bayesian inference system (BIS).

**FIGURE 7:**
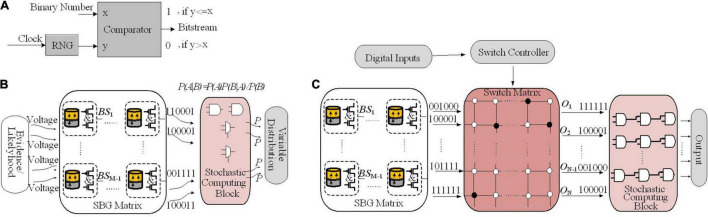
**(A)** Stochastic bitstream generator (SBG) block design. **(B)** Stochastic Bayesian inference system. **(C)** Spintronic-based Bayesian Inference System (SPINBIS) diagram work in this figure (switch matrix).

Figures 7A,B describe the diagram of the proposed BIS in Jia et al. (2018). A SBG block consists of a RNG and a comparator, which together generate the corresponding bit stream (Figure 7A). The input of BIS is shown in Figure 7B, which is a series of bias voltages proportional to evidence or likelihood. These evidences or likelihoods may come from sensors of different platforms. The SBG matrix and the SC architecture are two key components of a BIS. The SBG matrix is employed to translate the input voltages to stochastic bitstreams. The stochastic computing architecture is constructed by simple logic gates such as AND gate and scaled addition implemented by a multiplexer (MUX) and takes SBs as inputs. Stochastic computing block implements Bayesian inference by a novel arrangement of logic gates.

For an SBG, the small margin input voltages (Jia et al., 2018) is highly problematic when it generates the output probability. Digital-to-analog converters (DACs) with high precision are needed for precise mapping from digital probabilities to voltages. In addition, tackling the nonlinear relationship between probabilities and voltages is difficult and a slight noise or process variation may translate a probability to a wrong voltage value. In order to address these limitations, for the specified applications a prebuild SBG array utilizing SBG sharing strategy is employed. It is implemented by hybrid CMOS/MTJ technologies named spintronic-based Bayesian inference system (SPINBIS) (Figure 7C). The aim of proposing the SPINBIS is to enhance the stability of SBG and to use a smaller number of SBGs (Jia et al., 2020). The outcome probability of each SBG is predetermined and is then multiplexed through the switch matrix, which is a crossbar array. This crossbar array is constructed from transistors implemented at cross points, which are controlled by the switch controller. Since the SBG array is prebuilt, it should provide enough kinds of bitstreams to have an accurate stochastic computing block. In order to improve the energy efficiency and speed of SBG circuit, a state-aware self-control mechanism is utilized. Thanks to the SBG sharing property, the inputs with the same evidence can be modeled by the bitstream of the same SBG. However, for the inputs that are related together by one or more logic gates, which are called conflicting inputs, sharing the same SBG is problematic and is not allowed. The SBG sharing mechanism provides a much smaller number of SBGs compared with the input terminals of stochastic computing logic since the non-conflicting terminals are allowed to share the same bitstream. For data fusion applications, SPINBIS provides 12× less energy consumption compared to the MTJ-based approach (Jia et al., 2018) with 45% area overhead and about 26× compared to the FPGA-based implementation. On the other hand, the relation between probability and voltage is not very smooth; as a result, the stability of the proposed SBG needs improvement. Although the scale can be reduced, the switch matrix can show a congestion problem; hence, the reduction of the scale of SPINBIS is also worth exploring.

## Bayesian Inference Hardware Implementation With Digital Logic Gates

In this section, digital implementation of Bayesian inference will be discussed. First, we describe an implementation of Bayesian inference on HMM structures in digital logic gates. Next, an approximate inference algorithm based on a novel abstraction defined by stochastic logic circuits and some other hardware implementations of MRFs will be explained. Then, we describe C-Muller circuits as implemented with standard cells for Bayesian inference. Finally, we discuss probabilistic nodes based on CMOS technology. Hardware implementation of Bayesian inference employs the HMM network.

### Hardware Implementation of Bayesian Inference Employing Hidden Markov Model Network

**FIGURE 8:**
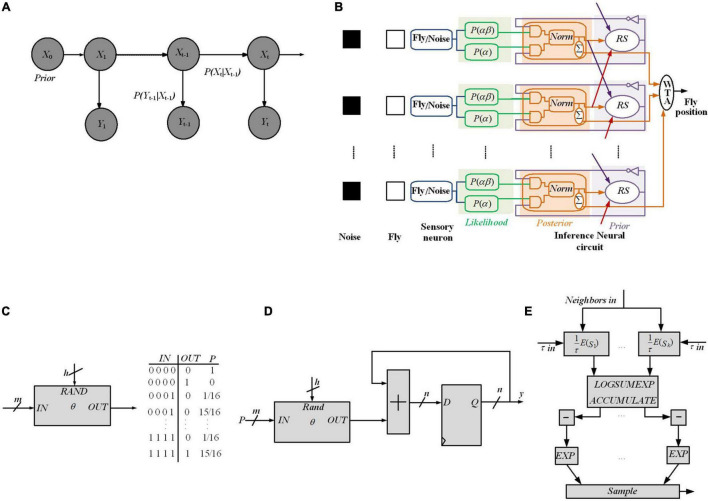
FPGA implementation of different Bayesian networks **(A)** An HMM architecture, in which the random variables *X*_t_ and *Y*_t_ are the hidden state at time *t* and the observation at time *t*, respectively (conditional dependencies are shown by arrows). **(B)** Digital Hardware implementation of HMM algorithm for Bayesian inference in Thakur et al. (2016). Implement the Gibbs sampling algorithms for Markov random fields in Mansinghka et al. (2008). **(C)** The schematic and CPT for a *O* gate, which flips a coin by specifying the weight on *IN* as a binary number [e.g., for *IN* = 0111, *P*(*OUT* = 1| *IN*) = 7/16]. A comparator that outputs 1 if RAND ≤ *IN* has been employed for implementing the *O* gate. **(D)** The proposed circuit for sampling, for a binomial distribution, utilizes *nh* bits of entropy to perform sampling while considering *n* flips of a coin of weight. **(E)** Gibbs pipeline, for Gibbs sampler depicting the required operations to numerically sample an arbitrary-size variable.

In Thakur et al. (2016), a hardware implementation of an HMM network has been proposed that utilizes sequential Monte Carlo (SMC) in SNNs. An HMM shown in Figure 8A models a system defined by a process that generates an observable sequence depending on the underlying process (Yu et al., 2018). In an HMM, *X*_t_ and *Y*_t_ represent the signal process and the observation, respectively. In a first order HMM, *Y*_*t*_, is considered as a noisy function of *X*_t_ and the development of a hidden state depends only on its current state. *X*_t_ is computed by its posterior distribution based on the noisy measurements or *Y*_t_. For a discrete-time problem, Equation (8) defines the first-order HMM, in which *d*_t_ and *v*_t_ denote random noise sequences.


(8) $${\text{X}}_{t}=\text{f}\left({\text{X}}_{t-1},{\text{d}}_{t}\right),{\text{Y}}_{t}=\text{g}\left({\text{X}}_{t},{\text{v}}_{t}\right)$$

The posterior density function *P* (*X*_t_| *Y*_1:t_) is computed recursively in two steps (i) prediction, and (ii) update. In the prediction step, the next state is estimated based on the current state utilizing the state transition model, without making use of new observations [see Equation (9)]. In contrast, the predicted state is updated utilizing the new observations at time *t* as shown in Equation (10).


(9) $$\text{P}\left({\text{X}}_{t}|{\text{Y}}_{1:t\text{-}1}\right)=\sum_{X_{t\text{-}1}}\text{P}\left({\text{X}}_{t}|{\text{X}}_{t\text{-}1}\right)\text{P}\left({\text{X}}_{t\text{-}1}|{\text{Y}}_{1:t\text{-}1}\right)$$

(10) $$\text{P}\left({\text{X}}_{t}|{\text{Y}}_{1:t}\right)=\frac{\text{P}\left({\text{Y}}_{t}|{\text{X}}_{t}\right)\text{P}\left({\text{X}}_{t}|{\text{Y}}_{1:t\text{-}1}\right)}{\sum_{X_{t}}\text{P}\left({\text{Y}}_{t}|{\text{X}}_{t}\right)\text{P}\left({\text{X}}_{t}|{\text{Y}}_{1:t-1}\right)}$$

In Thakur et al. (2016), to estimate a fly’s position at time t, a digital framework working based on the HMM rule shown in Figure 8B is utilized, through which a dragonfly tracks a fruit fly in a randomly flickering background. The sensory afferent neurons of the dragonfly fire probabilistically, when there is a fruit fiy or a false target (noise).

Dividing the state space (*X*_t_) into M discrete states reflects the fly’s (discretized) position at time t. A sensory neuron and an inference neuronal circuit demonstrate each discrete state. The fruit fly’s position at time t is predicted by the dragonfly’s central nervous system through utilizing the statistics of the output spikes of the sensory afferent neurons until time (t-1), and updates the prediction when it receives a new observation (*Y*_t_), at time t. Utilizing prediction and update Equations (9) and (10), it can be written as:


(11) $$P\left(X_{\text{t}}|Y_{1:t}\right)\propto P\left(Y_{\text{t}}|X_{\text{t}}\right)\sum_{X_{t-1}}P\left(X_{\text{t}}|X_{t-1}\right)P\left(X_{t-1}|Y_{1:t-1}\right)$$

where *P*(*Y*_t_ | *X*_t_) is the likelihood, *P*(*X*_t_ | *X*_t–1_) is the transition probability, and *P*(*X*_t–1_| *Y*_1:t–1_) is the posterior at the previous time step. Here, *Y* ∈ *R*^M^, and M denotes the total number of states. At each time step, for each state, the probability of the fly is computed. To this end, a WTA circuit is used to predict the fruit fly position by finding the maximum a posteriori of the probability distribution over states.

To predict the position of the fruit fly, the posterior probabilities of the state space is employed. To this end, an algorithm is utilized, which is similar to the SMC technique as a Monte Carlo method, useful for sequential Bayesian inference (Gordon, 1993). In the proposed framework, spikes denote a probability distribution over a set of states (i.e., the probability of a state is proportional to the sum of its spikes) and the RS (resampling) neuron block encodes the transition model, *P*(*X*_t_ | *X*_t–1_) through spatial connections (Figure 8B).

The likelihood generator block has a Poisson neuron (PN), generating spike trains based on its intrinsic firing rate, α and αβ (α: the probability of firing of the *k*th sensory neuron, either due to a fruit fly or a distractor; αβ is a spike when there is no fly, but a distractor instead). To implement the posterior generator block, the two subblocks of the Coincidence Detector (CD) neurons along with the normalization (norm) neural circuits have been utilized. Since the likelihood spike train does not depend on the prior spike train, a simple AND logic gate for the CD neuron can be utilized for the posterior implementation. The output spike trains of the CD neurons as the posterior probabilities of not having a fly and having a fly, respectively, are sent to the norm block to normalize spike trains.

Recurrent connection weights in the framework (shown by red, orange, and purple arrows in Figure 8B) are based on the transition probabilities. Spikes from the posterior distributions of adjacent norm neural circuits by considering their transition probabilities are sampled for a pathway by the RS block utilizing an inverse transform sampling approach in a discrete distribution.

Through collecting statistics of the spikes over many HMM time steps, the observation model parameters, α and αβ, are computed. At the start of the learning process, through stochastic exponential moving average filters (SEMAs), the parameters α and αβ are initialized and updated at each HMM time step for each location. An RNG circuit is implemented by the commonly used linear feedback shift register (LFSR) circuit. Neuronal building blocks used for implementing the HMM in Figure 8B are the PN, CD neuron, division, and normalization neural circuit, LFSR, and SEMA, which all are implemented on FPGA while all pathways are implemented in parallel on the FPGA hardware too. The implementation of these frameworks using simple logic gates will pave the way for stochastic computing to have digital hardware implementation of Bayesian inference using other approximation inference algorithms in spiking networks.

### Hardware Implementation of Approximate Inference Algorithm Using MCMC With Stochastic Logic Gates

By employing a novel abstraction, called combinational stochastic logic, probabilities are directly mapped to digital hardware in a massively parallel fashion (Mansinghka et al., 2008). On each work cycle, the output of a Boolean logic gate is a Boolean function of its inputs. Each gate represents a truth table whereas stochastic gates represent CPTs. Figure 8C shows the CPT and schematic for a gate called *O*, which generates flips of a weighted coin by specifying the weight on its input lines (*IN*) with h random bits on *RAND*. A comparator is utilized to implement the O gates where the output will be 1 if *RAND* ≤ *IN*.

Figure 8D shows a serial circuit composed of a stochastic logic gate, an accumulator, and a D flip-flop to implement the Gibbs sampling algorithms for MRFs. For a binomial distribution, this circuit utilizes *nh* bits of entropy to perform sampling while considering n flips of a coin of weight. It provides *O*(log(*n*)) space and *O*(*n*) time complexity. For a given variable, in order to implement a Gibbs MCMC kernel, a pipeline platform depicted in Figure 8E has been proposed (Mansinghka et al., 2008). Each possible setting while considering its neighbors under the joint density of the MRF has been scored by the pipeline and those scores have been tempered. Then, it computes the (log) normalizing constant and normalizes the energies. The normalized energies are translated to probabilities, and finally the pipeline outputs a sample. This pipeline can provide linear time complexity in the size of the variable by utilizing standard techniques and with a stochastic accumulator for sampling (using the circuit in Figure 8D). To this end, a fixed-point format is utilized to represent the state values, energies (i.e., unnormalized log probabilities), and probabilities. The logsumexp(*e*1,*e*2) function used for adding and normalizing the energies and the *exp*(*e*1) function used for converting the energies to probabilities are approximated. Then, the pipeline samples by exact accumulation. Moreover, numerically tempering a distribution, i.e., exponentiating it to some, can be utilized as energy bit shifting.

The proposed stochastic circuits have been implemented on Xilinx Spartan 3 family FPGAs. Typically large quantities of truly random bits are needed for stochastic circuit implementation. In almost all Monte Carlo simulations high quality pseudorandom numbers are used. For the FPGA implementation in Mansinghka et al. (2008), the XOR-SHIFT pRNG (Marsaglia, 2003) is used.

In order to develop more sophisticated circuits, such as circuits for approximate inference in hierarchical Bayesian models, which is a challenging research field, it is needed to combine the stochastic samplers with stack-structured memories and content-addressable memories (Shamsi et al., 2018; Guo et al., 2019). Moreover, directly using sub-parts from the proposed Gibbs pipeline to implement more sophisticated algorithms, including SMC methods and cluster techniques like Swendsen-Wang, is a promising research effort for the future.

There are a couple of works that provide MRF implementation for different applications *via* utilizing FPGA, application-specific integrated circuit (ASIC), graphics processor unit (GPU), and hybrid implementation *via* CPU+FPGA. Gibbs sampling as a probabilistic algorithm is utilized to solve problems represented by an MRF. In Gibbs sampling method, all random variables in MRF are iteratively explored and updated until converging to the final result (Bashizade et al., 2021). Ko and Rutenbar (2017) explores sound source separation while considering real-time execution and power constraints to isolate human voice from background noise on mobile phones. The implementation uses MRFs and Gibbs sampling inference, which demonstrates a real-time streaming FPGA implementation that achieves a speedup of 20× over a conventional software implementation. In addition, the approach also has a preliminary ASIC design-based implementation, which requires fewer than 10 million gates, with a power consumption of 52× better than an ARM Cortex-A9 software reference design. For more ASIC optimization, it is necessary to use a lower-power technology library and design optimization for lower memory usage.

In Seiler et al. (2009), an optimization framework utilizing a hierarchical Markov-random field (HMRF) implemented on a GPU is presented to deal with prediction/simulation of soft tissue deformations on medical image data. A method that combines mechanical concepts into a Bayesian optimization framework has been proposed (Seiler et al., 2009). This method has been implemented on a GPU and has been defined and solved under an HMRF approach. Providing an HMRF feature is an appealing technique that is able to solve the proposed stochastic problem since it was found that local minima are avoided. Where using a hierarchical approach and in addition, the nature of the hierarchical approach leads to a straightforward implementation in the GPU. It is assumed that the number of hierarchical levels on the number of iterations for the model to converge has a strong influence, which can be further explored in the future.

In Choi and Rutenbar (2013, 2016) to demand fast and high-quality stereo vision, a custom hardware-accelerated MRF system has been proposed for 3D gesture recognition and automotive navigation. The stereo task has been modeled as statistical inference on an MRF model and shows how to implement streaming tree-reweighted message-passing style inference at video rates. To provide the required speed, the stereo matching procedure has been partitioned between the CPU and the FPGAs. This partitioning provides using both function-level pipelining and frame-level parallelism. Experimental results show that this system is faster than several recent GPU implementations of similar stereo inference methods based on belief propagation.

As can be seen, there are still open windows to utilize new emerging nonvolatile devices and crossbar arrays to implement MRFs rather than just utilizing FPGA, ASIC, GPU, and hybrid implementations (CPU + FPGA). Moreover, refining the algorithms to make them more amenable to hardware implementations is needed while keeping the accuracy high.

### Muller C-Element Based Bayesian Inference

In order to calculate the probability of an event *V*, Bayesian inference incorporates the probability of V given the prior *P*(*V*) and evidence input *E*1 as in Equation (12), where, with parameter as defined by Equation (13), Equation (14) gets rewritten as Equation (15).

**FIGURE 9:**
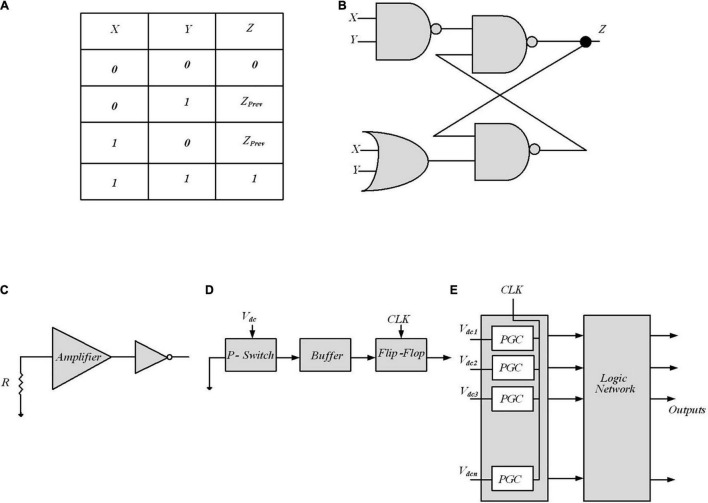
Digital implementation of Bayesian inference. **(A)** Muller C-element truth table in Friedman et al. (2016). **(B)** Standard cell C-element design of Muller C-element. PCMOS-based Bayesian network in Weijia et al. (2007). **(C)** p-switch circuit implementation block. **(D)** Probabilistic generating cells (PGCs) block. **(E)** The inference system utilizes a probabilistic generating block and a logic network.

The Muller C-element reported in Friedman et al. (2016), a two-input memory element, characterized by the truth table of Figure 9A, and shown in Figure 9B, performs the complete inference of Bayes’ rule. The output *Z* keeps its state, *Z*_prev_ while both inputs *X* and *Y* are opposite the current output state; afterward, it switches to the shared input value. A Muller C-element is able to compute Equation (14), thereby enabling efficient inference circuits. Note that input signals *i* with switching probabilities *a*_i_ and *b*_i_ for 0- > 1 and 1- > 0 switching, respectively, show no autocorrelation if *a*_i_ + *b*_*i*_ = 1. Then, considering no autocorrelation for input signals, the output probability is defined by Equation (15) for C-element, where P*(*E*1), *P*(*V*), and *P*(*V | E*1) are substituted by for *P*(*X*), *P*(*Y*), and *P*(*Z*). The reported Equation (15) is equivalent to Equation (14), representing the Bayesian inference provided by C-elements.


(12) $$\text{P}\left(\text{V}|\text{E}1\right)=\frac{\text{P}\left(\text{E}1|\text{V}\right)\text{P}\left(V\right)}{\text{P}\left(\text{E}1|\text{V}\right)\text{P}\left(\text{V}\right)+\text{P}\left(\text{E}1|\bar{\text{V}}\right)\text{P}\left(\bar{\text{V}}\right)}$$

(13) $${\text{P}}^{*}\left(E1\right)\equiv \frac{\text{P}\left(\text{E}1|\text{V}\right)}{\text{P}\left(\text{E}1|\text{V}\right)+\text{P}\left(\text{E}1|\bar{\text{V}}\right)}$$

(14) $$\text{P}\left(\text{V}|\text{E}1\right)=\frac{{\text{P}}^{*}\left(E1\right)\text{P}\left(V\right)}{{\text{P}}^{*}\left(E1\right)\text{P}\left(V\right)+\left(1-{\text{P}}^{*}\left(E1\right)\right)\left(1-\text{P}\left(V\right)\right)}$$

(15) $$\text{P}\left(Z\right)=\frac{\text{P}\left(X\right)\text{P}\left(Y\right)}{\text{P}\left(X\right)\text{P}\left(Y\right)+\left(1-\text{P}\left(X\right)\right)\left(1-\text{P}\left(Y\right)\right)}$$

Clocked bitstreams in stochastic computing are utilized to encode probabilistic signals permitting complex computations with minimal hardware and significantly improve the computation power consumption and inference speed when compared with conventional methods. Stochastic computing is not an exact computing technique and the slight loss of accuracy arises from several reasons. Compared to fixed or floating-point methods, in stochastic computing, the probability values *P* are usually translated to a stochastic bitstream with a lower quantization accuracy and the correlations between bitstreams usually lead to the loss of accuracy, since these bitstreams are usually generated by pseudo RNGs. Addressing this inherent imprecision and correlations need novel design techniques.

In Friedman et al. (2016), the number of “1”s in a bitstream encodes its probability and has nothing to do with the position of the 1 bits. In a stochastic bitstream, to represent a state switching probability a (b), i.e., the dynamics of a 0- > 1(1- > 0) switching, the probability R defined as R = a/(a+b). For an uncorrelated bitstream (i.e., a+b = 1), the probability is equivalent to R = a, where being “1” has a probability of R and being “0” has a probability of 1-R. Then, the switching rate for an uncorrelated bitstream is defined by Equation (16):


(16) S=2R(1-R)=2a(1-a)

The C-element outputs a stochastic bitstream, which is probabilistic and converging more slowly toward the exact Bayesian inference. If the switching rate of the output was low, the longer “domains” of consecutive “0”s and “1”s are needed and it leads to a more imprecise bitstream. Hence, more computation time is required to provide a precise output.

For multi-input Bayesian inference calculation, utilizing multi-stage C-element circuits is necessary, which would need one additional cycle per stage to compute a bitstream. On the other hand, the floating-point circuit provides a highly precise output while needing multiple pipelined computations and a long characteristic delay time. Hence, the C-element structure’s performance benefit is dependent on the required precision for the specific application.

For embedded decision circuits, where different independent sources of evidence are considered, for computing the probability of an event, C-element trees can provide direct stochastic hardware implementation. However, exploring the autocorrelation and inertia mitigation through signal randomization is required for further studies. For extreme inputs with low switching rates, the loss of accuracy is significantly increased. By increasing the length of the bitstream, the output signals converge in a polynomial manner to Bayesian precise inference. In addition, C-element trees have larger errors for opposing extreme input combinations. It is mentioned that this type of input and the error can be considered as strong conflicting evidence and the inference uncertainty, respectively.

The standard cells from Synopsys (Synopsys, 2012) SAED-EDK90-CORE library are used (Tziantzioulis et al., 2015) for C-Muller module implementation. For a two-input Bayesian inference implementation the standard cells have been employed and the simulation results showed that the floating-point circuit utilizes 16,000× area more than a C-element. This is due to the fact that for a two-input inference problem, just one C-element is required while the conventional floating-point circuit needs addition, multiplication, and division units. Also, for multi-input Bayesian inference, the C-element still outperforms the floating-point circuit.

### Probabilistic CMOS Based Bayesian Inference

In Weijia et al. (2007), probabilistic CMOS (PCMOS) technology has been used to implement RNGs to create a highly randomized bit sequence suitable for inference in a Bayesian network. A PCMOS-based RNG is composed of the PCMOS switch or p-switch, which is a CMOS switch with a noise source coupled at its input node. Figure 9C shows a p-switch block. The resistor is employed as a source of thermal noise, which follows the Gaussian distribution. An amplifier is used to amplify the noise signal to have a comparable signal with supply voltage.

The inference system is shown in Figure 9E, composed of probabilistic generating block and logic network. The probabilistic generating block generates random bits with different probabilities, and the logic network defines the edges between the nodes in a Bayesian network. The probabilistic generating block is composed of a number of probabilistic generating cells (PGCs), each of which generates a “1” bit with a probability. A PGC shown in Figure 9D is made up of a p-switch, a buffer, and a flip-flop. The buffer constructed from two inverters strengthens the output signal of the switch. The flip-flop, formed by two D latches, synchronizes the PGCs. Arithmetic operations (addition and multiplication in Bayesian network) computed in computers require a lot of time and energy. Here, two simple logic gates (an AND gate and an OR gate), together with some inverters, are employed to construct the logic network. To determine the approximate probability of the output at each node, a simulation has been performed to generate a 10,000-bits sequence at each node and then measure the “1”-bits in each sequence. The PCMOS-based hardware implementation of the Bayesian network outperforms the software counterpart in terms of energy consumption, performance, and quality of randomness. However, making use of mixed-signal implementations needs paying attention to noise and variation sources as well as examining the multiple independent sources of evidence for embedded decision circuits that require circuit design remedies.

## Crossbar Arrays for Bayesian Networks Implementation

In this section, two brain-inspired hardware implementations of inference in naïve Bayesian (NB) classifiers will be discussed. These implementations use memristors as nonvolatile elements for the inference algorithm implementation. Bayesian reasoning machine with magneto-tunneling junction-based Bayesian graph is explained.

### Crossbar Arrays for Naïve Bayesian Classifiers

**FIGURE 10:**
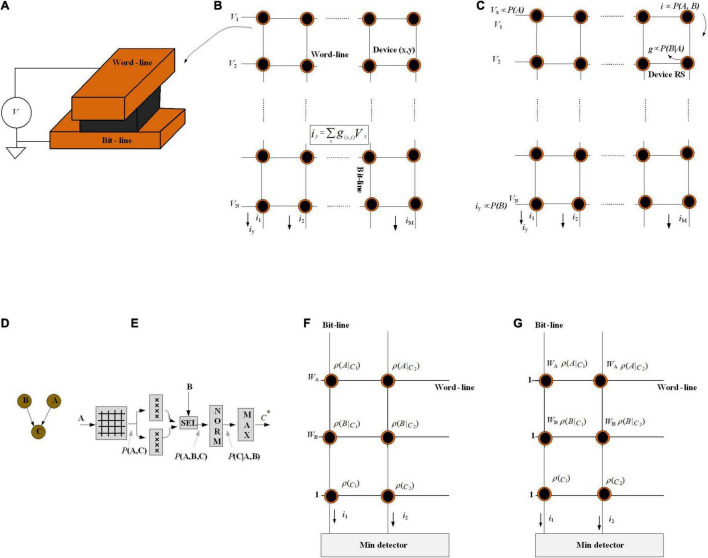
**(A)** Schematic of the memristor device in which the device’s active material is surrounded by two electrodes [top (wordline) and bottom (bitline)]. **(B)** Ohm’s law: *i* = *g*. *V* is utilized to perform multiplier operation. **(C)** The crossbar array is used as a Bayesian inference system. **(D)** Graphical model for Bayesian network. **(E)** Implementation of the Bayesian classifier. **(F)** Implementation of the naïve Bayesian classifier for a network of two attributes. **(G)** Implementation of a different way to calculate *I*_c_. In this method, the weight of the *i*th attribute (*w*_*i*_) is stored in the cell resistance.

A crossbar array of memristors is a promising hardware platform for Bayesian processing implementation in a massively parallel and energy-efficient way (Yang et al., 2020). Figure 10A depicts a schematic view of a memristor cell, in which a storage layer is sandwiched between the top and bottom electrodes, and the conductance of the device is dependent on the applied voltage. Figure 10B shows a crossbar array; it represents a maximum area efficiency of 4F^2^ per cell (Wu et al., 2019). Memristor crossbar arrays provide a natural implementation of matrix-vector multiplication (MVM). The current flowing through a memristor cell at the wordline *x* and bitline *y* is equal to *V*_*x*_*g*_(*x*,*y*)_. Here, *V*_*i*_ is the voltage applied to the wordline *x* and *g*(*x*, *y*) is the conduction of the cell. The total current through the bitline *y* is ∑_*x*_*V*_x_g(*x*, *y*), which implements a dot product of *V*_x_.g(*x*, *y*). The algorithmic complexity of MVM is reduced from O(*n*^2^) to O(1), which makes them a promising computing paradigm for different machine learning applications (Wu et al., 2019).

To perform Bayesian inference, Figure 10C shows a memristive crossbar array where a discrete distribution represented by a voltage is injected to the wordlines, the conditional probability *P*(B| A) translate to the memristor conductance, and all bit-lines are virtually earthed. Utilizing the current summing action of the crossbar bitlines, the current of each memristor is proportional to *P*(B| A)⋅*P*(A) = *P*(A, B), which is marginalized to *P*(B). Finally, inputs are multiplied by memristor conductances (*g*_k_) and exit as currents.

In analog systems, due to the noise, mismatch, and other variation sources, the input vectors do not necessarily meet the fact that the probability distributions of random variables must sum up to 1. To this end, the “normalizer” circuit is employed as a supporting module. Moreover, utilizing a linear method to convert the probability into voltage levels or memristor resistive states limits the dynamic range of the probability. That is, very small probability values may be translated into voltages below the noise levels in the system (Serb et al., 2017). However, the normalizers could scale these values when they are very low, but similar. It turns out to be problematic if there are very large probability values as well as very low ones in the same distribution. To solve this issue, it has been suggested that the resistive state/voltage needs to be mapped to the log probability domain (Wu et al., 2019).

Naïve Bayesian classifiers assume that the feature variables are all independent of each other (Serb et al., 2017) and the classification is based on the Bayesian theorem. For a test instance *x*, represented by an attribute value vector (*A, B*), the NB finds a class label *c* that provides the maximum conditional probability of *c* given *A, B*.

In Serb et al. (2017), a small graphical model for the prediction of potential health issues (Figure 10D) has been supposed to be implemented in memristor crossbar arrays, where *A* shows the air quality as *A* ∈ {bad, medium, good}, and B shows the corresponding heartbeat of the patient for two different activities *B* ∈ {resting, exercising}. Then, by considering random variables *A*, *B*, in order to predict the probability of a health crisis, and thus to clarify whether to warn the patient, i.e., *C* ∈ {safe, crisis} with a classic NB classifier, the goal of NB is to find a class label *c* that has the maximum conditional probability of *c* given *A, B* (as attributes):


(17) $$\begin{array}{c} {\text{C}}^{*}={\text{max}}_{C}\left\{\text{P}\left(\text{C}|A,B\right)\right\}\\ where\text{P}\left(\text{C}|A,B\right)\alpha \text{P}\left(\text{A}|\text{C}\right)\text{P}\left(\text{B}|\text{C}\right)\text{P}\left(C\right) \end{array}$$

C* is defines as the maximum *a posteriori* estimate.

Figure 10E depicts the hardware implementation process of the proposed NB framework. With air quality level A as input and the crisis level prediction C as an output, first, a crossbar stores *P*(*A*, *C*) = *P*(*A*| *C*)*P*(*C*), before factoring heart rate B in. Then, the output is sent in parallel to two arrays of memristors that maintain *P*(*B* = resting | *C*) and *P*(*B* = exercising | *C*), respectively. Based on the heartbeat *B*, one of the two outputs would be selected to put into the normalizer to calculate *P*(*C*| *A*, *B*). Finally, the max-finder module finds the estimate C*. This inference platform depicts that with the crossbar arrays as well as utilizing a cascade of small modules, it is able to scale to more complicated graphical models.

As discussed above, by directly employing the multiply accumulate capabilities of the crossbar array, the inference can be performed. During learning, as new data arrives, the conditional probability matrix needs to be updated; thus, the devices in the crossbar need to be programmed. The conductance stability and the energy efficiency of memristor switching, i.e., how many attempts are needed to reach the memristors desired state, determine the energy, speed, and circuit complexity cost of the probability updates (Serb et al., 2017). In Equation (17), it has been assumed, given the class, that all attributes (A, B) are fully independent of each other. The classification accuracy would be harmed when this assumption is violated in reality.

Wu et al. (2019) propose another analog crossbar computing architecture to implement the NB algorithm while considering the abovementioned concerns. It assigns every attribute a different weight to indicate different importance between each other. This assignment relaxes the conditional independence assumption. The prediction formula is formally defined as:


(18) $${\text{C}}^{*}\left(x\right)=\text{max}\left\{\text{P}\left(c\right)\text{P}{\left(\text{A}|\text{c}\right)}^{w_{A}}\text{P}{\left(\text{B}|\text{c}\right)}^{w_{B}}\right\},\text{c}\in \text{C}$$

where *w*_A_ and *w*_B_ are the weight of attributes A and B, respectively. The NB classifier in Equation (17) is a special case of the Weighted NB (WNB) classifier when *w*_A_ and *w*_B_ are equal to 1.

Naïve Bayesian formula [Equation (18)] transformation to the crossbar array Equation (18) cannot be directly applied to the Memristor crossbar array (concern 1). So, a log(•) operation is applied because *P*(•) ∈ (0, 1). log *P*(•) is a negative value that cannot be represented by the conductance of memristor cells as the conductance is always positive; then, ρ(•) denotes -log *P*(•) and then Equation (18) is rewritten as:


(19) $$\begin{array}{c} {\text{C}}^{*}\left(x\right)=\text{min}\left\{\rho \left(c\right)+{\text{w}}_{A}\cdot \rho \left(\text{A}|\text{c}\right)+{\text{w}}_{B}.\rho \left(\text{B}|\text{c}\right)\right\}\;\text{c}\in \text{C}\\ \text{q}\left(c\right)=\rho \left(c\right)+{\text{w}}_{A}.\rho \left(\text{A}|\text{c}\right)+{\text{w}}_{B}.\rho \left(\text{B}|\text{c}\right) \end{array}$$

The *q*(*c*) rewritten in the form of a dot product v^→^.g^→^, where v^→^ = [1 *w*_*A*_, *w*_*B*_ ] and g^→^ = [ρ(*c*), ρ(**A**|**c**), ρ(**B**|**c**)]. Hence, it is feasible to compute *q* of every class by the MVM.

After training, every prior probability ρ(*c*) is stored, as well as every conditional probability ρ(*A| c*) in the crossbar array in the form of memristor conductance, where *c ∈ C.*

For attribute A, voltage *w*_A_ is applied to the wordline (Figure 10F) and the current gathered on this sub-bitline (*I*^A^*_*c*_*). With the addition of ρ(*c*), the final result is obtained as current on one bitline. Multiple bitlines together give answers of Equation (19) to all classes. Optimization has also been proposed to the input voltage. Due to the *I*–*V* nonlinearity of the ReRAM cell, the analog input voltage (i.e., *w*_*i*_) might result in inaccuracy. The weight *w*_*i*_ is included in the cell conductance shown in Figure 10G.

The simulations show that the design offers a high runtime speedup up with negligible accuracy loss over the software-implemented NB classifier. This brain-inspired hardware implementation of NB algorithm as well as providing insights from techniques like mean-field approximation (Yu et al., 2020) will help to find an optimal balance between structure and independence, using hardware feasibility considerations and independence assumptions as mutually constraining objectives, which can be a promising research field.

### Bayesian Reasoning Machine With Magneto-Tunneling Junction-Based Bayesian Network

**FIGURE 11:**
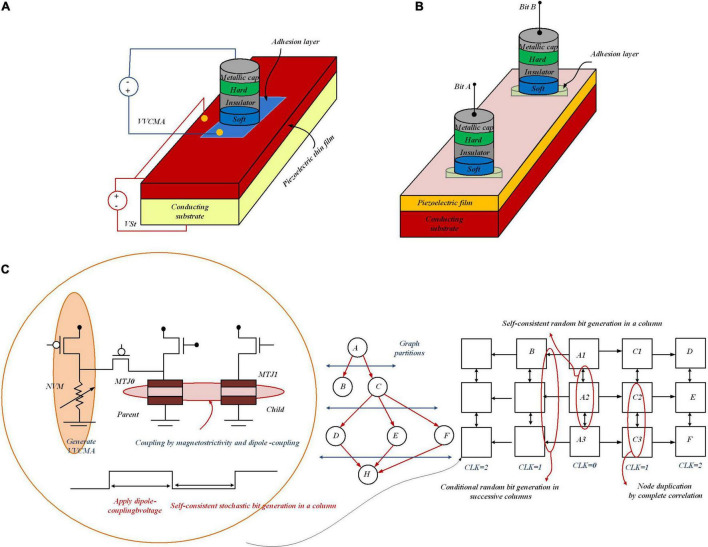
Stochastic random number generation utilizing MTJs with programmable probability. **(A)** MTJ with VCMA and STT (applied *via* the voltage *V*_VCMA_). For programmable random number generation, the magnetostriction, i.e., strain (controlled with the voltage *V*_St_) can be co-optimized. **(B)** MTJ integration utilizing the effect of dipole coupling (controlled with local stress applied to one MTJ) can be used to couple the switching probability of one MTJ depending on the state of the other. Thereby, the correlation between the bits “*A*” and “*B*” can be controlled *via* the resistance states of the two MTJs. **(C)** MTJ network-based Bayesian reasoning machine to show an example mapping of Bayesian graph on 2D nanomagnet grid.

Predictions from Bayesian networks can be accelerated by a computing substrate that allows high-speed sampling from the network. Nasrin et al. (2020) provide the development of such a platform to map an arbitrary Bayesian network through an architecture of the MTJ network along with circuits to writing, switching, and interactions among MTJs. By these means, electrically programmable sub-nanosecond probability sample generation, voltage-controlled magnetic anisotropy (VCMA), and spin-transfer torque (STT) have been co-optimized. As Figure 11A shows for programmable random number generation, VCMA, STT (applied *via* the voltage VCMA), and magnetostriction, i.e., strain (injected with the voltage *V*_*St*_), in an MTJ are co-optimized. To stochastically couple the switching probability of one MTJ depending on the state of the other, as Figure 11B depicts, MTJ integration is required, in which dipole coupling, controlled with local stress, is applied to one MTJ. This results in electrically tunable correlation between the bits “*A*” and “*B*” (encoded in the resistance states of the two MTJs), without requiring energy-inefficient hardware like OP-AMPS, gates, and shift-registers for correlation generation. To compute posterior and marginal probabilities in Bayesian networks *via* stochastic simulation methods, samples of random variables are drawn to determine the posterior probabilities. For the platform, mere stochasticity in devices is not enough, and for a scalable Bayesian network, “electrically programmable” stochasticity to encode arbitrary probability functions, *P*(*x*); *x* = 0 or 1, is required; moreover, this “electrically programmable” stochasticity is necessary for stochastic interaction among devices for conditional probability, *P*(*x*| *y*). In the presence of thermal noise at room temperature, the “flipping” is stochastic, i.e., the magnetization will precess when *V*_VCMA_ is turned on and can either return back to the original orientation or flip to the other orientation. By adjusting the magnitude of *V*_VCMA_, the probability of flipping can be tuned. Therefore, the voltage *V*_VCMA_ as a knob controls the probability of getting either “0” or “1.” The MTJ grid in Figure 11C only enables the nearest-neighbor correlation, and each node can only have binary states. For nodes with more than two states, splitting by binary coding is required. In order to run a general Bayesian network on the 2D grid, new mapping and graph partitioning/restructuring algorithms must be developed.

In Figure 11C, an example mapping strategy is shown to run general edges in a graph. Graph nodes are duplicated by setting the coupling voltages for perfect anti-correlation. To perform independent sampling on the MTJ grid, it is required to map the parent variables on the parent MTJ column and the children on the successive columns. In the stochastic simulation, different sampling algorithms on the grid are tested to speed up the process of sample generation of random variables in a Bayesian network to compute the posterior probabilities. These algorithms speed up the inference in Bayesian networks but can still fall short of the escalating pace and scale of Bayesian network-based decision engines in many Internet of Things (IoT) and cyber-physical systems (CPS). With a higher degree of process variability, prediction error for *P*(*F*) increases. By increasing the size of the components (resistive memory, current biasing transistor, etc.) as well as post-fabrication calibration, tolerance to process variability in the proposed design can be increased. The discussed platform would pave the way for a transformational advance in a novel powerful generation of ultra-energy-efficient computing paradigms, like stochastic programming and Bayesian deep learning.

## Bayesian Features in Neural Networks

In this section, employing Bayesian features in neural networks is represented. To this end, first Bayesian neural networks are explained. Then, Gaussian synapses for PNNs will be introduced. Afterward, a PNN with memristive crossbar circuits is described. At the end of this section, approximate computing to provide hardware-friendly PNNs and an application of probabilistic ANN for analyzing transistor process variation are explained.

### Bayesian Neural Networks

Bayesian deep networks define the synaptic weights with a sample drawn from a probability distribution (in most cases, Gaussian distributions) with learnt mean and variance and inference based on the sampled weights. In Malhotra et al. (2020), the gradual reset process and cycle-to-cycle resistance variation of oxide-based resistive random access memories (RRAMs) and memristors have been utilized to perform such a probabilistic sampling function.

Unlike standard deep networks, defining the network parameters as probability distributions in Bayesian deep networks allows characterizing the network outputs by an uncertainty measure (variance of the distribution), instead of just point estimates. These uncertainty considerations are necessary in autonomous agents for decision-making and self-assessment in the presence of continuous streaming data. In Bayesian formulation, defined by Equation (20), *P*(*W*) represents the prior probability of the latent variables before any data input to the network and *P*(*D*| *W*) is the likelihood, corresponding to the feedforward pass of the network. *P*(*W*| *D*) is the posterior probability density where two popular approaches, variational Bayes inference methods and Markov chain Monte Carlo methods, are used to make its estimation tractable.


(20) $$\text{P}\left(\text{W}|\text{D}\right)=\frac{\text{P}\left(\text{D}|\text{W}\right)\text{P}\left(\text{W}\right)}{\text{P}\left(D\right)}$$

In Malhotra et al. (2020) and Yang et al. (2020), the variational inference approach has been used since it is scalable to large-scale problems. In the variational inference approach, to approximate the posterior distribution, a Gaussian distribution, *q*(*W*, θ), is used. *q*(*W*, θ) is characterized by parameters, θ = (μ, σ) in which μ and σ, respectively, are the mean and standard deviation vectors for the probability distributions representing *P* (*W*| *D*) [see Equation (21)]. The main hardware design concerns for implementation of Bayesian neural networks are Gaussian random number generation block and dot-product operation between inputs and sampled synaptic weights.

**FIGURE 12:**
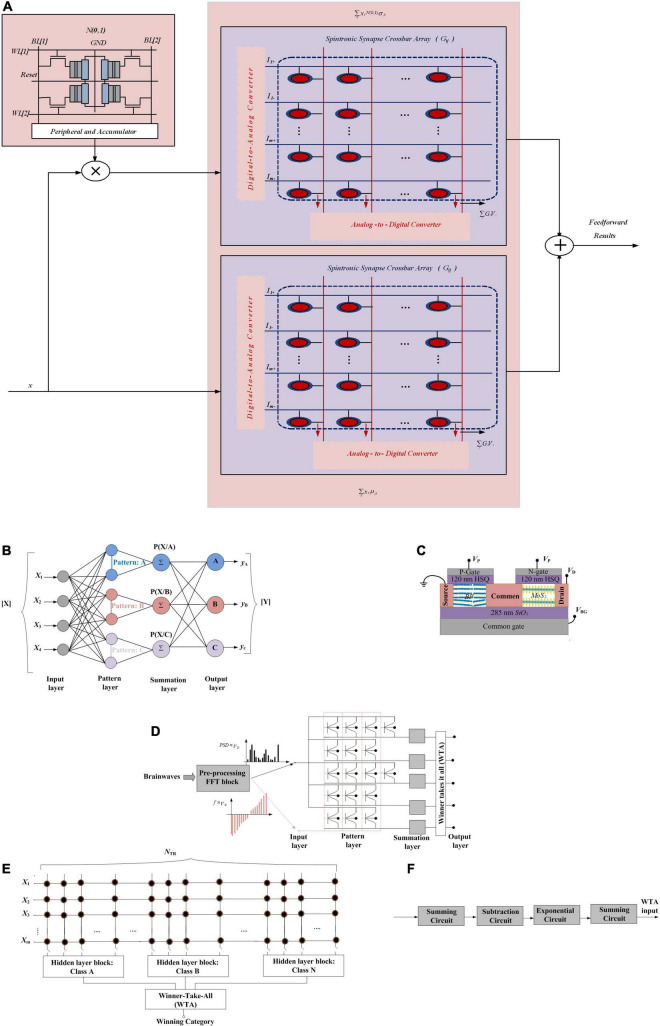
**(A)** All-spin Bayesian neural network implementation. The RNG unit performs sampling operation from Gaussian random number generators and the two crossbar arrays provide the “In-Memory” computing kernels (Malhotra et al., 2020). **(B)** The structure of the Probabilistic Neural Network (PNN) model (Gaussian Synapse based PNN) translates any input pattern to any number of output classifications through using a pattern layer and a summation layer. **(C)** Schematic of a reconfigurable Gaussian synapse composed of dual-gated n-type MoS_2_ and p-type black phosphorus (BP) back-gated field-effect transistors (FETs). Hydrogen silsesquioxane (HSQ) was used as the top-gate dielectric and nickel/gold (Ni/Au) was used as the top-gate electrode. **(D)** PNN Architecture for Brainwave recognition. The amplitude of the FFT data is passed from the input layer to the pattern layer as drain voltage (*V*_D_) of the Gaussian synapses, and the frequency range is mapped to the back-gate voltage (*V*_G_) range. The summation layer integrates the current over the full swing of *V*_G_ from the individual pattern blocks and communicates with the winner-takes-it-all (WTA) circuit and then the output layer recognizes the brainwave patterns. **(E)** The architecture of the PNN (Akhmetov and Pappachen, 2019) is implemented with crossbar arrays where each class has been implemented with a crossbar array where *N*_TR_ denotes the total size of the training set (classes). **(F)** Block diagram of the hidden layer block.

A Normal distribution with a particular mean and variance is equivalent to a scaled and shifted version of a Normal distribution with zero mean and unit variance. This consideration would allow partitioning the inference equation as shown in Equation (22). The _jk_, and σ_jk_ are the mean and variance of the probability distribution of the corresponding synaptic weight. As shown in Figure 12A, to construct the resultant system, the domain-wall MTJ memory devices based on two crossbar arrays are used for the _jk_ and σ_jk_ implementation, respectively. While the inputs of a particular layer are directly applied to the crossbar array storing the mean values, they are scaled by the random numbers generated from the RNG unit.

The output of the network, *y*, corresponding to input, *x*, is defined by Equation (21). As all the posterior distributions are learnt, the network output averages the outputs provided by sampling from the posterior distribution of the weights, *W*, where, *f*(*x*,*W*) is the network mapping for input *x* and weights, W.


(21) $$\text{y}={\text{E}}_{P\left(W|D\right)}\left[\text{f}\left(x,w\right)\right]\sim {\text{E}}_{q\left(w,\theta \right)}\left[\text{f}\left(x,w\right)\right]\sim \frac{1}{\text{S}}\sum_{i=1}^{s}\text{f}\left(x,{\text{w}}^{i}\right)$$

The approximation is done over S independent Monte Carlo samples from the Gaussian distribution, *q*(*W*,θ). *f*(*x*,*W*_*i*_) for the *j*th neuron can be decomposed into Equation (22), by considering just a single layer and neglecting the neural transfer function.


(22) $$\begin{array}{c} f\left(x,w_{ij}\right)=\sum_{k}x_{k}N\left(\mu_{jk},\sigma_{jk}\right)\\ \;=\sum_{k}x_{k}\cdot \left(\mu_{jk}+\sigma_{jk}.N\left(0,1\right)\right)\\ \;=\sum_{k}x_{k}\cdot \mu_{jk}\sum_{k}x_{k}.N\left(0,1\right)\sigma_{jk} \end{array}$$

The proposed “all-spin” Bayesian neural processor has the potential of providing orders of magnitude area, power, and energy consumption efficiency over the state-of-the-art CMOS implementations. A significant rethinking of the co-design space of device circuits and algorithms is necessary for Bayesian deep learning since it provides a unique computing framework that combines both deterministic (dot-product evaluations of sampled weights and inputs) and stochastic computations (sampling weights from probability distributions).

### Gaussian Synapse-Based Hardware Implementation for Probabilistic Neural Networks

In the computing revolution era, scaling in the semiconductor industry is inevitable and has three characteristic aspects: energy scaling, size scaling, and complexity scaling. Energy scaling satisfies the situation of the practically constant computational power budget. Through size scaling, more transistors can be fabricated in the same chip area, which consequently provides a faster and cheaper computing system. Complexity scaling ensures incessant growth in the computational power of a single on-chip processor. Considering these requirements, Sebastian et al. (2019) enable the hardware implementations of PNNs (shown in Figure 12B) *via* introducing a new class of analog devices, namely, the reconfigurable Gaussian synapses based on the heterostructure of atomically thin 2D layered semiconductors (shown in Figure 12C). The 2D materials satisfy aggressive size scaling while energy scaling is ensured *via* analog Gaussian synapses, and complexity scaling is met by PNNs. *Via* threshold engineering of the proposed device, it shows complete compatibility of amplitude, mean, and standard deviation of the Gaussian synapse. As shown in Figure 12B, unlike ANN, which employs multiple hidden layers with a large number of nodes in each layer, PNN proposed by Specht (1990) is a supervised learning neural network based on Bayesian decision rule and is composed of a pattern layer and a summation layer. PNNs are able to map any input pattern to any number of output classifications. Furthermore, in ANNs, activation functions such as sigmoid and rectified linear unit (ReLU) are used, where various derivatives of these functions have been utilized to determine the pattern statistics (which are extremely difficult for non-linear decision boundaries to perform with reasonable accuracy). In PNNs, parent probability distribution functions (PDFs) are used for the class probability. PDFs are approximated by a Parzen window and a non-parametric function, which is a Gaussian distribution for a Gaussian kernel (Specht, 1990). In PNNs, arbitrarily shaped decision boundaries are used, which facilitate the accurate classification of complex patterns. Moreover, since multivariate Gaussian kernels are simply generated from the product of univariate kernels, PNNs can be extended to map higher-dimensional functions. A reconfigurable Gaussian synapse, with dual-gated (DG) MoS_2_ and BP FETs, is shown in Figure 12C. Hydrogen silsesquioxane (HSQ) was used for the fabrication of the top-gate dielectric. Nickel/gold was used (Ni/Au) for the top-gate electrode fabrication for different top-gate voltages (*V*_N_). The back-gate threshold voltage (*V*_TN_ of the MoS_2_ FET) is tuned by *V*_N_. The top-gate voltage is tuned to control the height of the potential barrier for electron injection inside the MoS_2_ channel. Moreover, a back-gate voltage conducts current from the source to the drain terminal. The PNN architecture has been implemented on brainwave recording data, for each type of brainwaves. The frequency pattern of the normalized power spectral density (PSD) is extracted from the fast Fourier transform (FFT) of the time domain electroencephalography (EEG) data with increasing sampling times. As the training set becomes large, the discrete frequency responses of each type of brainwave evolve into continuous spectrums representing complex patterns. The functional dependence of the PSDs on the frequency makes the system highly nonlinear. Hence, using conventional ANNs can be challenging for the classification of brainwave patterns. To provide reasonable accuracy in ANNs, optimum training algorithms and extensive feature extraction and preprocessing of the training sample are required, while PNNs provide single-pass learning. This learning mechanism happens *via* defining the class PDF for each of the brainwave patterns in the frequency domain through employing the Gaussian mixture model (GMM). As described by Equation (23), GMM is represented as the weighted sum of a finite number of scaled (different variance) and shifted (different mean) normal distributions. ψ_I_ as component weights, μ_*i*_ as component means, and σ_*i*_^2^ as variances are for parameterizing a GMM with K components through which the total probability distribution must normalize to unity.


(23) $$\begin{array}{c} \text{P}\left(x\right)=\sum_{j=1}^{k}\psi_{i}\text{N}\left[{\frac{\text{x}}{\mu }}_{i},\sigma_{i}\right];\text{N}\left[\frac{\text{x}}{\mu_{i}},\sigma_{i}\right]\\ \;=\frac{1}{\sqrt{2\psi_{i}^{2}}}\cdot {\text{exp}}^{-\frac{{\left(x-\mu_{i}\right)}^{2}}{2\sigma_{i}^{2}}};\sum_{j=1}^{k}\psi_{i}=1 \end{array}$$

For each type of brainwave pattern, the GMM parameters for the K components are estimated based on the training data and utilizing the non-linear least square method. Root mean square errors (RMSEs) are calculated as a function of K. K denotes the number of Gaussian curves used in the corresponding GMMs. For each of the brainwaves, to define the non-linear decision boundary, a limited number of Gaussian functions are required. Hence, the energy and size constraints for the PNNs based on Gaussian synapses are enormously reduced. Finally, the PNN architecture shown in Figure 12D is evaluated for the detection of new brainwave patterns. The amplitude of the new FFT data in PNN (which consists of input, pattern, summation, and output layers) is passed as the drain voltage (*V*_D_) of the Gaussian synapses from the input layer to the pattern layer. The frequency range is translated to the back-gate voltage (*V*_G_) range. The summation layer collects the current over the full swing of *V*_G_ from the individual pattern blocks. After current integration in the summation layer, the currents communicate with the WTA circuit. The WTA detects the brainwave patterns in the output layer. It is shown that utilizing Gaussian synapses in PNN architecture can recognize complex neural oscillations and brainwave patterns from a large number of EEG data providing extreme energy efficiency, which will foster the feasibility of efficient hardware implementation of PNNs and subsequently high-performance and low-power computing paradigm.

### Probabilistic Neural Network With Memristive Crossbar Circuits

Probabilistic neural network architecture (Specht, 1990) provides a fast training mechanism in which weights are derived from training samples directly and set in the first initialization stage. Then, the density functions of the categories are estimated based on the training dataset. The input samples are classified based on these density functions. PNNs provide the ability to converge to Bayes optimal decision surface without trapping to local minima. Moreover, a new training pattern can be added to the network that does not require any global retraining process. On the other hand, for hardware implementation of the near-edge computing devices, the processing speed, the size of the network, and the power consumption are critical. PNN’s parallel computational nature and fast learning PNNs make them attractive for hardware implementation and utilization in near-edge computing devices. In Akhmetov and Pappachen (2019), a hardware implementation of the PNNs based on the memristive (ReRAM based) crossbar architecture has been proposed (shown in Figure 12E); to this end, a crossbar with *N*_TR_ dimensions is utilized to perform dot product between weights of the pattern neurons and input vector, where *N*_TR_ denotes the total size of the training set. The proposed circuit provides the density estimation and classification performed by the PNN. As shown in Figure 12B, the input layer of the PNN distributes an input to pattern neurons. The pattern layer performs a dot-product operation and exponential activation. The summation neurons integrate the outputs of pattern neurons belonging to one class and then in the output layer the decision is made. In the output layer, the density functions are scaled by their prior probability and loss function; after that, the category with the highest posterior probability is chosen as the output of the PNN. The hidden layer block shown in Figure 12E computes the approximate density functions of categories based on the training set and is composed of summing circuits, a subtraction circuit, and the exponential function generator (Figure 12F). The sub-blocks of the hidden layer block are implemented with CMOS circuits. The system-level simulation showed that the proposed implementation of the PNN is insusceptible to process variation of the ReRAM and provides a high accuracy on the MNIST dataset. Future studies should implement a memristor programming circuit (to provide on-chip learning), employ alternative kernel functions and ReRAM devices, and utilize a larger dataset.

### Approximate Computing to Provide Hardware Friendly Probabilistic Neural Networks

Approximate computing greatly improves computing in computer systems *via* accomplishing more tasks under the same resource consumption. On the other hand, a large number of floating-point operations and multipliers are required in DSP hardware architectures needing a large number of hardware resources. Although by using fixed-point arithmetic implemented in hardware the DSP algorithm can process the constant multiplication simultaneously, this can reduce the accuracy of the calculation. To solve these hardware circuit design problems, the PNN hardware architecture of approximate calculation using a genetic algorithm (GA) has been proposed in Chen et al. (2019). GA realizes approximate calculation of the hardware circuit of PNN, to achieve the best balance between maintaining good classification ability and the least hardware resource consumption to reduce the hardware complexity. The key concept of GAs is to imitate the natural evolution law of natural selection in nature and to solve the optimization problem utilizing three main operators: reproduction, crossover, and mutation. Firstly, one encodes all the parameters into chromosomes, and defines a fitness function. The evolution starts from the population of completely random individuals, evaluates the adaptability of each chromosome to the environment in each iteration process, and then generates the new population through natural selection and mutation. This is to be repeated until the final break conditions are met. The hidden layer neurons of PNNs (shown in Figure 12B) are responsible for the computer rate density function, which performs the nonlinear transformation from the input space to the hidden layer. The weight vector of hidden layer neurons represents a training pattern, and the probability density function is a Gaussian function in multidimensional feature space, which is a nonlinear function. Such nonlinear functions are often implemented on hardware. In addition, the Gaussian function is decided by a smoothing coefficient of its distribution scope σ. The larger σ, the wider the breadth, and the smaller σ, the narrower the breadth. If the input vector is located close to the center of the Gaussian function, the hidden layer node will generate a larger output. In practical engineering, the look-up table is often used to approximate these nonlinear functions. In Chen et al. (2019), the number of bits encoded by the smoothing parameters and probability values of a PNN is used as the gene encodes each individual in GA, and the recognition rate of the PNN classifier is used as the fitness function, using GA to optimize the parameters to obtain the circuit structure with both the correct rate and the low memory resource consumption. While ensuring that the correct rate is not affected, GA is used to search for the optimal parameters of the PNN and establish a look-up table method for nonlinear functions to simplify the complexity of the hardware architecture, reduce the use of logic gates (the Altera MAX 10 device was used for simulation), and increase the operation speed. This work provides new insights to utilize evolutionary algorithms in a Bayesian computing platform to optimize the rules and consequently improve the hardware efficiency.

### Probabilistic Artificial Neural Network for Analyzing Transistor Process Variation

Line-edge-roughness (LER) is a process-induced random variation source that causes undesirable random variation in the performance of transistors such as metal oxide semiconductor field effect transistor (MOSFET), fin-shaped field effect transistor (FinFET), and gate-all-around field effect transistor (GAAFET). LER can be analyzed with technology computer-aided design (TCAD), which is fundamentally very time-consuming. A machine learning-based method to solve this issue is proposed in Lim et al. (2021), which predicts the LER variations in FinFETs, through which LER parameters (i.e., amplitude and correlation length X, Y) are provided as inputs for an ANN. ANN predicts seven parameters: off-state leakage current (Ioff), saturation drain current (Idsat), linear drain current (Idlin), low drain current (Idlo), high drain current (Idhi), saturation threshold voltage (Vtsat), and linear threshold voltage (Vtlin). To this end, a 3-D quasi atomistic model for LER was used. FinFET was simulated with MATLAB and TCAD by applying the mentioned parameters and the two-dimensional autocovariance function. Considering that the performance metrics of transistors approximately follow Gaussian distribution is not applicable due to non-ideal effects (short-channel effects in transistors) and the different distribution shapes for each LER parameter. Hence, the mixture of multivariate normal distributions (MVN) is used during the training process. Negative log-likelihood (Negloglik) was used as a loss function [see Equation (24)] instead of mean-squared error since, during the training, the weight matrices and bias vectors of ANN are updated for the given layer attached to output neurons returning the PDF of variables. The training process is run to minimize this loss function; hence, training ANN becomes the process of maximum likelihood estimation.


(24) $$\text{Negloglik}\left(P,Q\right)=-\sum_{k}\text{P}\left(x\right)\text{logQ}\left(x\right)$$

In Equation (24), *P*(*x*) and *Q*(*x*) stand for the PDF of observation and hypothesis, respectively. The proposed ANN models have reduced the simulation time by ∼6 times and can pave a new road to analyzing the impact of LER to overcome the timely design process *via* simulating the electrical behavior of the transistor as well as DC behavior of critical digital circuit blocks in processors such as SRAM bit cells.

## Hardware Implementation of Probabilistic Spiking Neural Networks

In this section, employing Bayesian features in SNN is represented, in which the feasibility of nonvolatile devices as synapses in SNN architectures will be first discussed for Bayesian-based inference algorithms. Then, a scalable sampling-based probabilistic inference platform with spiking networks is explained. Afterward, a probabilistic spiking neural computing platform with MTJs is explained. The high learning capability of a probabilistic spiking neural network implementation and utilization of the probabilistic spike propagation mechanism are described. At the end of this section, memristor-based stochastic neurons for probabilistic computing and Loihi-based Bayesian inference implementation are discussed.

### Bayesian Inference Implementation in Spiking Neural Networks With Memristor Synapses

Memristors are another type of nonvolatile (i.e., the device could save its state when there is no voltage source) memory devices. They are promising circuit elements that mimic the functionality of biological synapses in a neuromorphic computing system (W. Burr et al., 2017). Their resistance can be tuned based on the spike-timing dependent plasticity (STDP) rule, which is based on the spike timing differences of the pre- and postsynaptic neurons. There are practical challenges in the fabrication of reliable nanoscale memristors. In order to address these challenges, an alternative approach is proposed to use the compound memristive synapse model, where *M* bistable memristors in parallel model a synapse (Bill and Legenstein, 2014) with a total weight of:


(25) $${\text{W}}_{\mathrm{ki}}=\omega .{\text{m}}_{\mathrm{ki}}$$

**FIGURE 13:**
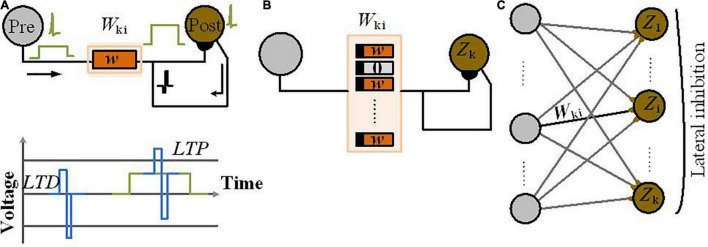
**(A)** STDP pulsing scheme along with pre- and postsynaptic spikes behavior for LTP and LTD phenomena. **(B)** Stochastic memristors construct the compound memristive synapse. **(C)** The winner-take-all mechanism in SNN with compound memristive synapses.

A compound synapse provides *M*+ 1 discrete weight levels from 0 to the maximum level *W*_max_ = ω⋅*M*, where *m*_ki_ ∈ {0, 1,…, *M*} in Equation (26) represents the number of active memristors. The weight change of the compound memristive synapse is controlled by pre- and postsynaptic activity. An input pulse (Figure 13A) of the *i*th input is defined by *y*_i_(t) = 1 [and no presynaptic pulse by *y*_i_(t) = 0] and *t*^fk^ denotes the spike time of the *f*^th^ spike of postsynaptic neuron *Z*_k_. A neuron *Z*_k_ generates a spike train *S*_k_(t) represented as the sum of Dirac delta pulses δ(⋅) at the spike times: *S*_k_(t) = ∑_*f*_δ(*t*−*t*_*fk*_). When a synaptic *W*_ki_ is subject to a stochastic long-term potentiation (LTP), where the presynaptic neuron spikes before the postsynaptic neuron, there are (*M* - *m*_ki_) inactive memristors (Figure 13B). Each memristor independently turns into its active state with probability π_up_, hence contributing ω to the *W*_ki_. Thereby, the weight change for the LTP condition is equal to (*M* - *m*_ki_).ω.π_up_. A similar argumentation applies to the long-term depression (LTD) case, where the post neuron spikes first (before the presynaptic neuron). Note that LTP (LTD) occurs when the presynaptic pulse equals *y*_i_(t) = 1 [*y*_i_(t) = 0], respectively. Then, the weight change of the compound memristive synapse is:


(26) $$\begin{array}{c} <\frac{\text{d}}{\text{dt}}{\text{W}}_{\mathrm{ki}}>\\ ={\text{S}}_{k\left(t\right)}\cdot [\underset{\mathrm{LTP}}{\underbrace{\left(\text{M}-{\text{m}}_{\mathrm{ki}}\right)\omega \pi_{\mathrm{up}}{\text{y}}_{i}\left(t\right)}}-\underset{\mathrm{LTD}}{\underbrace{{\text{m}}_{\mathrm{ki}}\omega \pi_{\mathrm{down}\left(1-y_{i}\left(t\right)\right)]}}} \end{array}$$

Compound memristive synapses with the STDP property have been employed in winner-take-all (WTA) (Wang et al., 2019) networks to provide stochastic learning capability from a Bayesian perspective as an unsupervised model optimization with the expectation-maximization method (Bill and Legenstein, 2014). As shown in Figure 13C, *N* spiking input neurons, *y*_1_,…, *y*_N_, and *K* spiking network, *Z*_1_,…, *Z*_K_, construct the WTA network. In the WTA network, the forward synapses provide all-to-all connectivity and the network neurons perform lateral inhibition in which the network neurons are competing with each other to fire.

Network neuron *Z*_k_, with the membrane potential *u*_k_, integrates the inputs *y*_i_(t) and the linear membrane potential can be implemented with leaky integrators (a common neuron model in neuromorphic computing paradigm). The neurons *Z*_k_ have a stochastic firing rate ρ_k_(t) and spike in a Poissonian manner. ρ_k_(t) defined by Equation (27), is a function of the membrane potential *u*_k_(t) and lateral inhibition *u*_inh_(t).


(27) $$\rho_{k\left(t\right)}={\text{r}}_{\mathrm{net}}\cdot {\text{e}}^{u_{k\left(t\right)}-u_{\mathrm{inh}\left(t\right)}}$$

The r_*net*_ constant scales the overall firing rate of the network. The lateral inhibition contribution *u*_*inh*_(t) : = log $\int_{j=1}^{k}\exp\left(U_{j}\left(t\right)\right)$ depicts WTA competition among the network neurons to fire over a given stimulus *y*_1_ (t),…, *y*_N_ (t).

When one of the network neurons, *Z*_k_, fires, the probability distribution *P*_net_(Z | Y) represents the network response that is proportional to the firing rate ρ_*k*_(t) of neuron *Z*_k_:


(28) $$\begin{array}{c} {\text{P}}_{\mathrm{net}}\left({\text{Z}}_{k=1}|\text{Y}=\text{y}\left(t\right)\right)=\frac{\rho_{k\left(t\right)}}{{\text{r}}_{\mathrm{net}}}\\ \;={\text{e}}_{k\left(t\right)}^{u}-{\text{u}}_{\mathrm{inh}}\left(t\right)=\sum_{j=1}^{k}{\text{e}}^{b_{j\left(t\right)}+\sum_{i=1}^{N}W_{\mathrm{ij}}\cdot y_{i\left(t\right)}} \end{array}$$

Claiming that the response distribution *P*_net_(*Z* | *Y*) provides a Bayesian performance is valid, by considering input *y*(t) as the observation variable and the spike response of a neuron *Z*_k_ as the hidden cause. The network is viewed as a generative model with a prior distribution *P*(*Z*) over hidden causes *Z*_k_ and a set of likelihood distributions *P*(*Y* | *Z*_k_ = 1), one for each hidden cause *Z*_k_.

Maximum likelihood learning finds parameters that bring the implicit distribution *P*(*Y*) of the generated model as close as possible to the actually observed input distribution. Likelihood distributions *P*(*Y* | *Z*_k_ = 1) optimized by a WTA circuit with compound-synapse STDP are computed through the product of the likelihoods of individual inputs:


(29) $$\text{P}\left({\text{Y}}_{i}=\text{y}\left(\text{t}\right)|{\text{Z}}_{k}=1\right)=\prod_{i=1}^{N}\text{P}\left({\text{Y}}_{i}={\text{y}}_{i}\left(\text{t}\right)|{\text{Z}}_{k=1}\right)$$

where the likelihood for each individual input *y*_i_ is represented by a Gaussian distribution:


(30) $$\text{P}\left({\text{Y}}_{i}={\text{y}}_{i}\left(\text{t}\right)|{\text{Z}}_{k}=1\right)=\frac{1}{{\sqrt{2\pi \sigma }}^{2}}\cdot e^{-\frac{{\left(y_{i(t)}-\mu_{ki}\right)}^{2}}{2\sigma }}$$

For the likelihood distributions, μ_ki_ and σ are the mean values and the standard deviation, respectively, and are identified as:


(31) $$\mu_{\mathrm{ki}}=\frac{{\text{W}}_{\mathrm{ki}}}{{\text{W}}_{\max}}=\frac{{\text{m}}_{\mathrm{ki}}}{\text{M}}\mathrm{and}\sigma =1/\sqrt{{\text{W}}_{\max}}$$

During online learning, a Mixture of Gaussians (MoG) generative model has been depicted by this probabilistic model of the WTA network, where compound memristive synapses show synaptic weight changes. This property on average causes the lower bound of the log-likelihood function to increase and leads to finding a local optimum. After training, Bayesian inference for the hidden causes based on the given input observation is employed; simulations have shown that even only four bistable memristors per synapse are sufficient for applications such as reliable image classification.

In hardware implementations of the WTA network, capacitors and other circuit elements have been used to implement the stochastic neurons, while synaptic weights are represented by conductance of compound memristors.

There are several challenges in this approach, mentioned below, which need further study:

- More complex inputs and plasticity mechanisms are needed to support a versatile STDP pulsing scheme; to this end, utilizing memristors with more than two stable states are required.
- Other arbitrary patterns of the input signal (*y*(t)) for compound memristor synapses are required to depict a clear picture of the Gaussian likelihood distributions *P*(*Y* | *Z*), which has the capability of performing inference over arbitrary real-valued input states.
- In compound memristor synapses, the switching probability (π_up_⋅*W*_max_) could be considered as the learning rate during online learning. This learning rate controls the number of samples of the input history of the implicit generative model. The number of samples is dependent on the size of the dataset. When the dataset is complex, it relies on small learning rates, i.e., on small switching probabilities, To achieve sufficiently small switching probabilities, it needs some remedies in hardware integration by using control peripherals.

### Scalable Sampling-Based Probabilistic Inference With Spiking Networks

The BrainScaleS platform (Schemmel et al., 2010), a physical-model neuromorphic device, emulates networks of spiking neurons. This platform is a mixed-signal neuromorphic system, using 180-nm CMOS technology for fabrication, on which Kungl et al. (2019) proposed the first scalable implementation of sampling-based probabilistic inference with spiking networks. In order to sample from target distributions and hierarchical spiking networks with higher-dimensional input data, fully connected spiking networks have been trained. Similar to systems that operate in biological real time, it provides a higher acceleration factor that shows the advantages of brain-inspired physical computation and maintain main building blocks for large-scale neuromorphic applications. Moreover, by co-embedding the stochasticity within the same substrate, the feasibility of a fully embedded neural sampling model with highly reduced demands on off-substrate I/O bandwidth has been shown, where having a fully embedded implementation allows the runtime of the experiments to scale as O(1) with the size of the emulated network.

The most notable limitation of the BrainScaleS system for this application was the size of the emulated spiking sampling network (SSNs). The maximum connectivity is limited (synapse loss) between different locations within the area, due to limited software flexibility, system assembly, and substrate yield; hence the applicable hardware real-estate was limited to a patchy and non-contiguous area. In order to write analog parameters, significant trial-to-trial variability for any given trail is needed, which leads to a heterogeneous substrate and a low sampling accuracy. The ability of the SSN to approximate target distributions has been hindered since the symmetry in the effective weight matrix is imperfect (due to analog variability of the synaptic circuits) and the resolution of the synaptic weights is low. Hence the “jumping” behavior between approximate and target distribution in the final stages of learning has been seen. Moreover, as the underlying neuron and synapse are deterministic, for a more biologically plausible implementation, one needs to consider stochastic neurons such that the framework can be extended to sampling from arbitrary probability distributions rather than only binary random variables.

### Probabilistic Spiking Neural Computing Platform With Magnetic Tunnel Junctions

In Sengupta et al. (2016), by enabling the neural computing unit with the stochastic switching behavior of an MTJ, the implementation of a deep SNN has been explored for high-accuracy and low-latency classification tasks and provided an energy improvement of 20× over a baseline CMOS design in 45-nm technology. Despite the huge success at complex recognition problems due to the high computational costs needed for training and testing of deep ANNs, researchers are motivated to develop alternative computing models; therefore, more biologically realistic SNNs have been introduced. In SNNs, information is transferred between the neural nodes as spikes rather than real-valued analog signals. Spiking networks exploit the prospects of event-based computing which lead to the development of specialized custom hardware implementations. Sengupta et al. (2016) discusses that the technologically mature spintronic devices, such as the MTJ (being binary switching devices), with variation in the magnitude of the input current showing switching probability variation similar to the sigmoid function. An ANN-to-SNN conversion scheme has been proposed utilizing the sigmoid function like switching probability of MTJs, and assuming that the neural units generate spikes depending on a probability density function (similar to the original ANN transfer function). It has been proved that such a conversion mechanism approximates the original ANN functionality to a reasonable degree of precision, potentially paving the way for probabilistic neuromorphic platforms that employ the variability and inherent stochasticity of emerging neuromagnetic devices. Nonvolatile emerging devices based on a probabilistic neural computing platform that models complex neural transfer functions in the time domain provide high-accuracy energy-efficient cognitive recognition platforms over conventional CMOS designs.

### High Learning Capability Probabilistic Spiking Neural Network Implementation

Using sequential processors to run algorithms, there is a struggle to simultaneously fulfill learning speed, learning performance, power consumption, and area requirements in portable and biomedical applications. Hence, hardware-implemented neural networks are used extensively and even though the circuit is implemented using analog very-large-scale integration (VLSI), variations in sensor fabrication, background noise, and human-dependent parameters complicate the restrictions on power consumption and area. One type of neural network that comprises spiking neurons with probabilistic parameters is called the probabilistic spiking neural network (PSNN). These PSNNs are hardware-friendly and compare with deterministic neural networks in hardware compatibility. PSNNs have relaxed weight resolution requirements and are insensitive to noise and analog process variation. A PSNN does not suffer from multiplicative linearity. In the spiking neuron model, the presynaptic spike of a neuron can be considered as a control signal, and the weight controls the postsynaptic current. As a result, when a presynaptic spike stimulates a neuron, the post synapse generates a current. In Hsieh et al. (2018, 2017), an analog implementation of PSNNs has been proposed for biomedical applications through which online learning adjusts weights by spike-based computation. The weight is saved in the long-term synaptic memory. Switched capacitor circuit structures have been utilized for the implementation of most of the circuits to provide low-power consumption and a small area and consequently provide high learning performance. This learning chip was fabricated in 0.18-μm CMOS technology and can process the e-nose and electrocardiography (ECG) data, yielding comparable accuracy to the simulated accuracy that indicates that the learning chip can be employed into portable and implantable devices, to facilitate convenient use and intelligence. This hardware implementation opens up new windows to achieve efficient portable and biomedical devices *via* utilizing PSNNs.

### Hardware Implementation of Spiking Neural Networks Utilizing Probabilistic Spike Propagation

**FIGURE 14:**
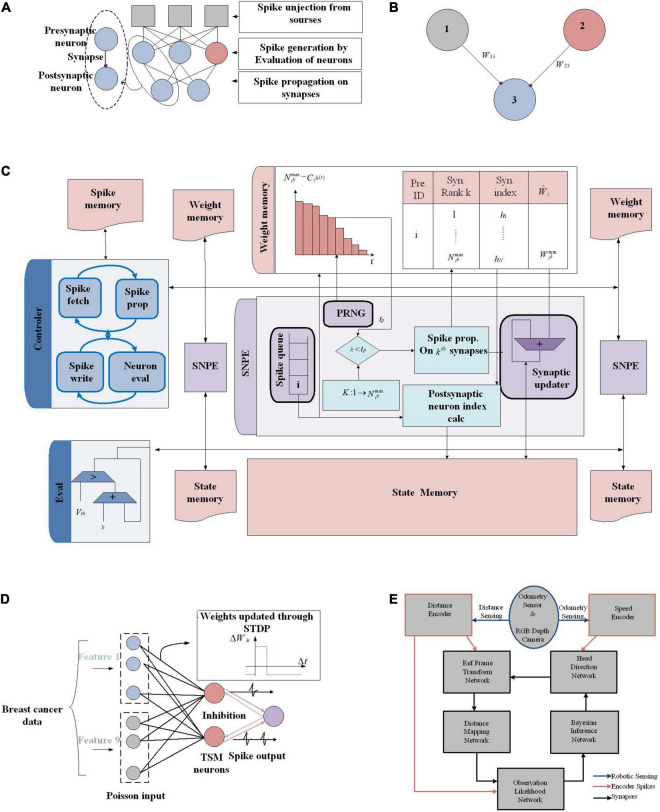
**(A)** A generic structure of spiking neural networks. **(B)** Neuron 3 receives spikes from Neuron 1 and Neuron 2. **(C)** P-SNNAP accelerator architecture in Nallathambi et al. (2021). **(D)** Stochastic neuron based probabilistic spiking neural network implantation for the uncertainty quantification problem in medical diagnosis (Wang et al., 2021). The probabilistic SNN consists of the input encoding layer, the output layer, and the inhibitory layer. Neurons mimic the biological neurons behavior. Each feature of breast cancer data is encoded by the firing rate of a small population of Poisson neurons. The inset depicts the STDP learning curve of synapses connecting input neurons to the output ones; if the post-spike falls within the time window after the pre-spike, then the synaptic potentiation will occur. **(E)** Overall structure of the implemented SNN architecture on the Loihi processor (Tang et al., 2019).

As mentioned in *Section “*Bayesian Inference Implementation in Spiking Neural Networks With Memristor Synapses,” SNNs provide intrinsic desirable attributes where information is represented as discrete spike events that provide an event-driven paradigm of computation. SNNs are implemented on low-power event-driven hardware, and the time and energy consumption are proportional to the number of spike events. When processing a spike, SNNs do not require multiplication to be performed and hence provide a reduced hardware complexity compared to conventional ANNs; as a result, SNNs are not well-suited to be implemented on hardware platforms like GPUs. Spiking networks still need a large number of memory accesses although they are event-driven. It is necessary to know the fanout neurons of a spiking neuron, which determines the connectivity information that needs to be fetched along with the weights of the corresponding synapses. Then, the membrane potentials of the fanout neurons are fetched and updated. Defining techniques for reducing the number of memory accesses in SNNs is necessary for improving their energy efficiency since data fetching from memory is more expensive than arithmetic computations. The spiking activity that is measured as spike propagation along a synapse from a single source neuron to a single target neuron has a strong role in the complexity of an SNN. Nallathambi et al. (2021) introduce an approach that is named probabilistic spike propagation to optimize rate-coded SNNs. In this approach, synaptic weights are represented as probabilities, and these probabilities are utilized to regulate spike propagation. The approach reduces the propagated spikes, which cause a reduction in time and energy consumption. To this end, an SNN accelerator named probabilistic spiking neural network application processor (P-SNNAP), which supports probabilistic spike propagation, has been represented, where a probabilistic method for spike propagation to reduce the number of memory accesses in rate-coded SNNs has been proposed. This method would save both runtime and energy. The proposed probabilistic spike propagation mechanism has been realized through probabilistic synapses shown in Figures 14A,B. Conventionally, the weight of a synapse determines the amount by which the potential of postsynaptic neuron membranes increases whenever presynaptic neuron spikes. This weight defines how likely it is that a spike will propagate across the synapse (Figures 14A,B). A probabilistic synapse does not propagate all spikes to the postsynaptic neuron. Instead, only a subset of its outgoing synapses propagate the spike (which has weights above a certain threshold) as neuron spikes. P-SNNAP is shown in Figure 14C. The P-SNNAP architecture consists of three different modules, the Spike Neural Processing Element (SNPE), the Eval unit, postsynaptic spikes, the weight memory that stores the weights, and the state memory that stores the neuronal state variables. The Eval unit performs neuron evaluation. Eval unit brings membrane potentials from state memory, increases it with bias value, and compares it to the threshold. If the membrane potential goes above the threshold, a spike is generated and communicated to the controller. The controller in its first phase of operation controls the SNPEs and that in the second phase controls the Eval unit. When a layer is evaluated by Eval, the controller brings spikes from the previous layer and sends them to SNPEs. As a spike is received, an SNPE uses the index of the spiking neuron to iterate through its outgoing synapses. The SNPE calculates the index of the postsynaptic neuron for each synapse. Next, the membrane potential and the weight of the corresponding synapse are fetched for each postsynaptic neuron. Then, membrane potential is updated and written back. All the information that is required to perform probabilistic spike propagation is stored in weight memory in each SNPE lane. The register transfer level was used for the P-SNNAP engine design and the Nangate 15-nm technology was used for synthesizing in Synopsys Design Compiler platform. It has been observed that the proposed probabilistic approach causes a logic area and logic power overhead of 12 and 23.5%, respectively, over a version of SNNAP without support for probabilistic spike propagation. In this work, it has been shown that the temporal nature of SNNs allows the network to regain any accuracy loss caused by this approach. Evaluating alternative synaptic propagation mechanisms and employing larger networks to test the scalability of the proposed accelerator turn out to be further explored.

### Memristor-Based Stochastic Neurons for Probabilistic Computing

Stochastic firing mechanism in biological neurons (rather than giving out spikes once reaching a fixed threshold voltage) provides dynamic excitation behavior and allows the brain to perform probabilistic inference in the face of uncertainty. However, due to the complexity of the stochastic firing process, fabrication of stochastic neurons with bio-realistic dynamics to probabilistic scenarios is challenging and needs further study. In Wang et al. (2021), a stochastic neuron has been fabricated based on CuS/GeSe threshold switching memristor (TSM) and applied to implement Bayesian computing in a PSNN that can quantify uncertainty with incomplete or inaccurate data. The experimental results have indicated that compared to VO_2_ and GeTe_6_, which are typical metal-insulator transition (MIT) and ovonic threshold switching (OTS), CuS/GeSe as a conductive-bridge TSM shows the most appropriate randomness of threshold switching as desired by the stochastic firing of neurons. The proposed physical modeling and simulation have revealed that this can be attributed to the similarity between the ion motion tuning in conductive-bridge threshold switching and in biological neurons. In particular, the positive feedback process of Cu electromigration enhanced Joule heating and temperature and thereby accelerated thermal diffusion of Cu, substantially facilitating the formation of the conductive bridge and the stochasticity of ion motion, which leads to the desired variation of threshold voltages. The intrinsic random formation of the Cu conductive bridge in the device is utilized to emulate the stochasticity of the opening of ion channels in the biological membrane. Moreover, the random switching parameters of the device fulfill the requirement to achieve the stochastic neurons in a PSNN. Utilizing the stochastic firing properties of the fabricated CuS/GeSe neuron to a probabilistic SNN is shown in Figure 14D. This probabilistic SNN is capable of giving superior prediction on a typical probabilistic inference problem, namely, breast cancer diagnosis with high diagnostic accuracy, and improves the fidelity of the judgment compared to deterministic neuron-based SNN. Moreover, the stochastic neurons enable the SNN to estimate the uncertainty of predictions, a feature that will be of great help for achieving a good balance between diagnostic accuracy and medical cost and avoiding the fatal diagnostic misclassification error often encountered by conventional ANNs. The software synapses used in this demonstration can be achieved by non-volatile memristors, indicating the possibility of implementing a fully memristive probabilistic SNN in the near future. Utilizing developed and optimized stochastic neurons and their powerful application in uncertainty quantification problems open up a new horizon of probabilistic computing in neuromorphic computing systems.

### Loihi-Based Bayesian Inference Implementation

Through asynchronous computations and event-based communications in a network of neurons, the brain solves simultaneous localization and mapping (SLAM) while it consumes very low energy; as Tang et al. (2019) show, SNNs (which are famous for mimicking this computational paradigm of the brain) can be used to solve SLAM problems on energy-efficient neuromorphic hardware for mobile robots exploring unknown environments. The proposed SNN shown in Figure 14E is integrated into Intel’s Loihi neuromorphic processor fabricated on 14-nm FinFET technology (Davies et al., 2018). Loihi is a non-Von Neumann hardware mimicking the brain’s computing paradigm and is optimized for SNN computations and online learning algorithms (Thakur et al., 2018).

*Via* multisensory cues (called visual and odometry information) to implement spike-based recursive Bayesian inference, Tang et al. (2019) proposed a model to determine the robot’s heading. To perform head direction localization and mapping, the recursive SNN suggests a cue-integration connectome on Loihi. The head direction and border cells in the network provide biologically realistic performance; thus, to implement them, the proposed model utilizes spiking neurons, multi-compartmental dendritic trees, and plastic synapses, each of which is implementable by Loihi. The model has two sensory spike rate encoders and five Bayesian networks. The odometry sensor drives the neural activity of speed cells, which encodes the angular speed and the RGB Depth camera drives the neural activity of sensory neurons, which encodes the distance to the nearest object. The head direction (HD) network defines the heading of the robot *via* receiving the input from the speed cells. The reference frame transformation (RFT) network generates allocentric distance representation *via* the HD network by getting its input from sensory neurons. The RFT network sends the allocentric observations to the distance mapping (DM) network and the DM network develops the map of the robot’s surrounding environment. The DM network sends its information to the observation likelihood (OL) network, which calculates the observation likelihood distribution of the robot’s heading. The Bayesian inference network through the utilization of the observation likelihood from the OL network and the odometry likelihood from the HD network provides an optimal posterior of the robot’s heading and corrects the heading representation within the HD network.

Note that each one of the networks is implemented on Loihi; here, the Bayesian inference network block is explained based on Equation (32).


(32) P(s|d,o)αP(d|s)P(o|s)P(s)

where *s*, *d*, and *o* denote the heading of the robot, the observed distance, and the odometry sensing, respectively. With a flat prior *P*(*s*), the posterior distribution over the robot’s heading is proportional to the product of *P*(*d*| *s*) and *P*(*o*| *s*), the two likelihood functions.

Having known that multiplying two Gaussian distributions generates another Gaussian distribution, Tang et al. (2019) have employed likelihood distributions represented by the OL network and the HD network to predict the posterior distribution. Dendritic trees have been used for implementation; specifically, each Bayesian neuron has two dendritic compartments connected with its corresponding OL neuron and HD cell.

Results of Loihi-based SNN architecture implementation show that it consumes 100 times less energy than conventional GMapping (a common algorithm for SLAM solving) running on a CPU. This provides a motivation to use Loihi as a hardware implementation platform for Bayesian inference. The FinFET technology used in the Loihi architecture is a promising technology in terms of energy and speed over conventional CMOS technology (Bagheriye et al., 2016), while the use of emerging nonvolatile technologies attracts a lot of attention to developing ultra-low energy computing platforms for SNN-based Bayesian inference systems (like crossbar arrays discussed in *Section “Crossbar Arrays for Bayesian Networks Implementation”*). However, the fabrication of robust nonvolatile devices and large-scale crossbar arrays probably require a lot more insights before they can outperform already highly developed technology and this approach is worth exploring.

## Discussion

In this paper, we have attempted to review and summarize the recent hardware developments for Bayesian inference. The review is centered on different possible hardware implementations considering algorithmic aspects. Different approaches and their principles have been discussed with extensive references quoted. We review the pros and cons of the approaches reported in the literature. Specifically, there are a number of challenges to be further studied before valid and robust models can be applied to practical systems. We summarize them as follows.

- In asynchronous implementation of Bayesian networks with spintronic devices, updating the network as well as dealing with variations in the thermal barriers or interconnect delays necessitates further study.
- In abstraction layer-based implementations based on the number of linearly independent equations, the appropriate number of auxiliary variables is needed; it would be challenging for a large Bayesian network and would add extra area and energy overhead, which requires further investigation.
- More complex inputs and plasticity mechanisms are needed to support a versatile STDP pulsing scheme *via* using memristors with more than two stable states as synapses to have biologically plausible Bayesian inference in SNNs. Other arbitrary patterns of the input signal for memristor synapses in SNNs is required to depict the clear picture of the Gaussian likelihood distributions that have a capability of performing inference over arbitrary real-valued input states. In memristor synapses, in SNN, the switching probability considered as the learning rate during online learning must be controllable since, for complex datasets, small learning rates, i.e., small switching probabilities, are required. Small switching probabilities need careful remedies in hardware integration by using control peripherals.
- In analog neuromorphic substrates like the BrainScaleS platform, due to limited software flexibility, system assembly, and substrate yield, the maximum connectivity between different locations is strongly limited; hence, post-production, assembly, and the mapping and routing software needed careful consideration to enhance on-wafer connectivity and thereby automatically increase the size of emulable networks, as the architecture of the SSNs. Moreover, approximation of the target distributions is hindered due to the limited synaptic weight resolution and the imperfect symmetry in the weight matrix (due to analog variability of the synaptic circuits). As a result of the successor system, a new generation of scalable platforms is needed to be designed with a higher weight resolution.
- Providing accurate digital encoding where Bayesian network representation is mapped directly (without any abstraction layer) to S-MTJ resistance with equivalent digital voltage representation using arithmetic composers is promising, whereas PSL needs an abstraction level to map Bayesian networks in hardware. To this end, using accurate encoding is required to achieve the required resolution.
- For the structure learning process of Bayesian learning, hardware acceleration *via* FPGA like system implementation is promising, since the runtime for Bayesian network inference has been highly reduced. This property attracts more attention to structure learning acceleration and could be a promising field to be studied utilizing emerging nonvolatile devices.
- In digital encoding of probabilities, the small margin input voltage is highly problematic when it generates the output probability. DACs with high precision are needed for precise mapping from digital probabilities to voltages. In addition, tackling the nonlinear relationship between probabilities and voltages is difficult and a slight noise or process variation may translate a probability to a wrong voltage value. The relation between probability and voltage is not very smooth as a result of the stability of the SBG, which needs improvement. Although the scale of hardware can be reduced, the reduction of the scale of the Bayesian inference system is also worth exploring. In addition, the resolution is limited since every storage method adds a resolution limitation; to this end, utilizing nonvolatile nanomagnets is promising to overcome the power consumption as well as increasing weight resolution by multi-state memristors as synapses.
- The C-element (a standard cell-based implementation) outputs a stochastic bitstream, which is probabilistic and converging more slowly toward the exact Bayesian inference. In this case, if the switching rate of the output was low, the longer “domains” of consecutive “0”s and “1”s are needed and it leads to a more imprecise bitstream and adds time, energy, and area overhead. On the other hand, mixed-signal implementations needs to pay attention to noise and variation sources as well as examining the multiple independent sources of evidence for embedded decision circuits that require circuit design remedies.
- Constructing circuits for approximate inference in hierarchical Bayesian models is a challenging research field that can be *via* merging stochastic samplers with stack-structured memories and content-addressable memories.
- The brain-inspired hardware implementation of algorithms like NB algorithm provides insights for techniques like mean-field approximation, which will help to find an optimal balance between structure and independence, using hardware feasibility considerations and independence assumptions as mutually constraining objectives, which can be a promising research field.
- To provide high-speed stochastic simulations (in which samples of random variables in a Bayesian network are drawn to determine the posterior probabilities), a variety of algorithms with higher sampling efficiency in Bayesian graphs are required since they still fall short of the escalating pace and scale of Bayesian network-based decision engines in many IoT and CPS.
- While providing conditional probability between two variables *via* utilizing two MTJs (or other nonvolatile devices) in a common substrate, the spacing between the MTJs needs to be carefully defined to have significant dipole coupling between the two soft layers. Moreover, in the presence of thermal noise at room temperature, the “flipping” is stochastic, which needs to be controlled with peripheral circuit elements and accurate timing.
- With a higher degree of process variability, prediction error for the probability of a variable is high. Tolerance to process variability needs to be increased by circuit innovations as well as post-fabrication calibration. Adapting ultra-energy-efficient nanomagnetic devices to stochastic/probabilistic computing, neuromorphic, belief networks (non-Boolean computing and information processing) has resulted in rapid strides in new computing paradigms, especially Bayesian networks that may experience revolutionary advances.
- In autonomous systems like self-driving cars, decision-making is based on uncertainty; hence, employing AI platforms is crucial. The standard supervised backpropagation-based learning techniques do not represent uncertainty in the modeling process to solve this issue; Bayesian deep learning plethora is required where a probabilistic framework following the classic rules of probability, i.e., Bayes’ theorem, has been utilized to train the DNNs.
- In a standard deep learning architecture during the inference, the dot-product operation between the synaptic weights and inputs involves the compute energy along with memory access and memory leakage components. In a Bayesian deep network, each synaptic weight uses double memory storage since it is represented by two parameters (mean and variance of the probability distribution). Moreover, the dot-product operation does not occur directly between the inputs and these parameters since for each inference operation the synaptic weights are repeatedly updated depending on sampled values from the Gaussian probability distribution. Hence, direct utilization of crossbar-based “In-Memory” computing platforms utilizing non-volatile memory technologies for mitigating the memory access, leakage, and memory fetch bottlenecks is not feasible; thus, a significant rethinking is necessary.
- Despite the specialized custom hardware and brain-inspired possibility of SNNs due to their event-based computing feature, their training for recognition problems has been mostly limited to single-layered networks. On the other hand, Bayesian techniques are more computationally expensive, thereby limiting their training and deployment in resource-constrained environments. Also, the standard von-Neumann bottleneck in current deep learning networks (where memory access and memory leakage can account for a significant portion of the total energy consumption profile) motivates further research in hardware implementation of multi-layer probabilistic SNN and is a promising research field.
- In Bayesian neural networks, Gaussian random number generation operation is a hardware expensive task for CMOS-based designs since a large number of registers, linear feedback circuits, etc. are required. To overcome this issue, non-idealities and stochasticity prevalent in RRAM, spintronic, and other nonvolatile technologies could be extensively exploited to this end.
- Stochasticity provides computational features like regularization and Monte Carlo sampling in a DNN where such normalization features reduce internal covariate shift obtaining an alternative process for divisive normalization in bio-inspired neural networks. Hence, employing the inherent weight normalization feature exhibited by a stochastic neural network using nonvolatile devices is a promising field where it is an online alternative for used batch normalization and dropout techniques. Saturation at the boundaries of fixed range weight formats as well as spurious fluctuations affecting the rows of the weight matrix have been mitigated.
- Despite the widespread applications and simplicity of PNNs, their hardware implementation is relatively underrepresented. This is due to the fact that multicomponent digital CMOS circuits that cause severe area and energy inefficiency are required for hardware implementation of probability functions associated with the PNNs, such as the Gaussian. Hence, utilizing emerging nonvolatile technologies to make use of their biological plasticity features of conductance level changes as well as their energy efficiency would be a promising solution that needs to be extensively explored.
- Uncertainty serves as an intrinsic part of neural computation through which probabilistic computing empowers the brain to analyze sensory stimuli, produce adequate motor control, and make reasonable inferences. On the other hand, quantifying uncertainty is especially crucial for error-critical applications like medical diagnostics, which require probabilistic SNN-based neuromorphic computing systems. The recent literature of electronic neurons for SNN implantation is mostly focused on deterministic neural units or emulating the complex biological neuronal functions, ignoring the demand of building intrinsically stochastic neurons. Hence, developing probabilistic spiking neurons with low area and power consumption is highly required.
- To foster the neuromorphic computing systems, not only is the mature device fabrication process required but also hardware friendly algorithms are inevitable, To this end, one promising approach that needs further exploration is utilizing evolutionary algorithms in a Bayesian computing platform to optimize the rules.

To conclude these observations, we can state that the pace of the development of efficient hardware implementation of Bayesian networks has been very quick in recent years, but there is still a long way to go to overcome the challenges outlined above. To summarize, comparing the discussed implementations shows that probabilistic hardware-based implementation of Bayesian networks, with nonvolatile devices, needs more attention to solving the scaling issues in Bayesian network hardware; also, sequential signaling from parent to child nodes, controlling the stochastic switching variation due to thermal noise and process variation, defining an abstraction layer, utilizing axillary nodes, employing complex input pattern for memristor synapse, and multi-state memristors for WTA mechanism are required. For NSMs for approximate Bayesian inference, providing technologically mature nonvolatile devices to solve the scaling issue in crossbar arrays on one hand and adding noise to provide uncertainty on the other hand are challenging tasks. Utilizing nonvolatile memory elements for Bayesian network implementation *via* digital encoding needs a high-resolution encoding mechanism to provide readily highly scaled FPGA-like architectures not only for inference but also for learning Bayesian network structure. To this end, utilizing multi-state memristors rather than two-state spintronic-based devices would provide higher resolution with a lower area overhead.

Bayesian inference hardware implementation employing digital logic gates in state-of-the-art FPGA platforms, defining novel stochastic logic gates, and utilizing standard cells needs to solve the accuracy and resolution issue of digital bitstreams, which needs to compromise speed, power, and area overhead. Crossbar arrays for Bayesian network implementation require some innovation where providing a hierarchy of crossbar arrays for approximate Bayesian inference mechanisms like mean-field approximation, taking inspiration from naïve Bayesian classifiers, is promising. Crossbar arrays require solving scaling issues while they act as Bayesian reasoning machines. Utilizing crossbar arrays in PNNs for dot-product operation needs serious rethinking while utilizing approximate inference rules. Moreover, utilizing platforms like BrainScaleS or Loihi is another option for Bayesian inference while the resolution and scaling, as well as energy consumption, need to be considered since these platforms are utilizing mixed-signal CMOS and FinFET technologies, respectively, rather than energy-efficient nonvolatile technologies.

## Conclusion

A Bayesian network provides a simple way of applying Bayes theorem to complex problems and Bayesian inference is crucial for statistical machine learning, causal discovery, automatic speech recognition, email spam filtering, and clinical decision support systems, to name just a few applications in AI. However, Bayesian inference is an NP-hard problem even when only an approximate solution is sought, implying that this computational problem scales badly, which hinders further progress in AI. Interestingly, many neuroscientists are convinced that our brains employ similar processes to combine prior knowledge with newly arriving information in an approximately optimal Bayesian fashion. For example, in visual perception, the brain establishes this integration literally in the blink of an eye. However, the brain’s energy consumption is orders of magnitudes less than what is required for state-of-the-art AI applications. Bayesian network implementations in conventional processor architectures are problematic due to several issues: (i) software solutions involve multiple layers of abstraction to support a non-deterministic framework such as Bayesian networks; (ii) the inherently separated memory and computation in the von Neumann processor architecture introduces bottlenecks in accessing data; and (iii) the non-volatility requirements in cognitive applications are challenging to meet the efficiency. Moreover, as mentioned, the computational complexity of belief updating is an important issue in Bayesian inference. To enhance the computation speed of Bayesian updating, several techniques such as conjugate priors, variational Bayes, or approximate Bayesian computations have been employed, whereas these are software-based, and their efficacy is less than hardware-based accelerators. Hence, the practical use of Bayesian inference has been hindered in many real-world applications (such as large-scale networks or embedded systems) where computational cost is an important performance factor. This review paper has discussed several implementations of Bayesian inference as well as the implantation of several approximate inference algorithms and different architectures, from FPGA-like to brain-inspired ones (crossbar arrays). FPGA-like architectures are not efficient enough in terms of area and energy overhead when compared to brain-inspired architectures. Crossbar arrays, a typical brain-inspired computing paradigm, lead to efficient computation when the network structure is limited to, e.g., naive Bayes classifiers or tree-like structures. Using insights into Bayesian approximation techniques to find an optimal balance between structure and independence and using hardware feasibility considerations and independence assumptions as mutually constraining objectives are open windows for future efforts to achieve an efficient computing paradigm.

## Author Contributions

LB and JK conceived the study and the discussions around material of the manuscript. LB wrote the first version of the manuscript then it has been edited by LB and JK. Both authors contributed to the article and approved the submitted version.

## Conflict of Interest

The authors declare that the research was conducted in the absence of any commercial or financial relationships that could be construed as a potential conflict of interest.

## Publisher’s Note

All claims expressed in this article are solely those of the authors and do not necessarily represent those of their affiliated organizations, or those of the publisher, the editors and the reviewers. Any product that may be evaluated in this article, or claim that may be made by its manufacturer, is not guaranteed or endorsed by the publisher.
